# Estimating Movement Smoothness From Inertial Measurement Units

**DOI:** 10.3389/fbioe.2020.558771

**Published:** 2021-01-14

**Authors:** Alejandro Melendez-Calderon, Camila Shirota, Sivakumar Balasubramanian

**Affiliations:** ^1^Cereneo Advanced Rehabilitation Institute (CARINg), Vitznau, Switzerland; ^2^Biomedical Engineering Group, School of Information Technology and Electrical Engineering, The University of Queensland, St. Lucia, QLD, Australia; ^3^Department of Physical Medicine and Rehabilitation, Northwestern University, Chicago, IL, United States; ^4^The Hopkins Centre, Menzies Health Institute Queensland, Griffith University, Nathan, QLD, Australia; ^5^Department of Health Sciences and Technology, ETH Zurich, Zurich, Switzerland; ^6^Department of Neurology, University of Zurich, Zurich, Switzerland; ^7^Department of Bioengineering, Christian Medical College, Vellore, India

**Keywords:** movement smoothness, inertial measurement units, movement kinematics, assessment, jerk, SPARC

## Abstract

Inertial measurement units (IMUs) are increasingly used to estimate movement quality and quantity to the infer the nature of motor behavior. The current literature contains several attempts to estimate movement smoothness using data from IMUs, many of which assume that the translational and rotational kinematics measured by IMUs can be directly used with the smoothness measures spectral arc length (SPARC) and log dimensionless jerk (LDLJ-V). However, there has been no investigation of the validity of these approaches. In this paper, we systematically evaluate the use of these measures on the kinematics measured by IMUs. We show that: (a) SPARC and LDLJ-V are valid measures of smoothness only when used with velocity; (b) SPARC and LDLJ-V applied on translational velocity reconstructed from IMU is highly error prone due to drift caused by integration of reconstruction errors; (c) SPARC can be applied directly on rotational velocities measured by a gyroscope, but LDLJ-V can be error prone. For discrete translational movements, we propose a modified version of the LDLJ-V measure, which can be applied to acceleration data (LDLJ-A). We evaluate the performance of these measures using simulated and experimental data. We demonstrate that the accuracy of LDLJ-A depends on the time profile of IMU orientation reconstruction error. Finally, we provide recommendations for how to appropriately apply these measures in practice under different scenarios, and highlight various factors to be aware of when performing smoothness analysis using IMU data.

## 1. Introduction

Inertial Measurement Units (IMUs) are becoming ubiquitous in everyday objects we carry, e.g., smartphones, smart watches, smart clothing, etc. This, along with the availability of relatively inexpensive IMUs, has sparked their use for movement analysis in different disciplines, such as movement neuroscience (e.g., Shull et al., 2014; Picerno, 2017a; O'Reilly et al., 2018), movement biomechanics and sports science (e.g., Li et al., 2016; Salmond et al., 2017; Johnston et al., 2019), and neurorehabilitation (e.g., Dobkin, 2013; Hubble et al., 2015; Vienne et al., 2017; Wang et al., 2017; Brognara et al., 2019; Parker et al., 2020).

**Figure 1:**
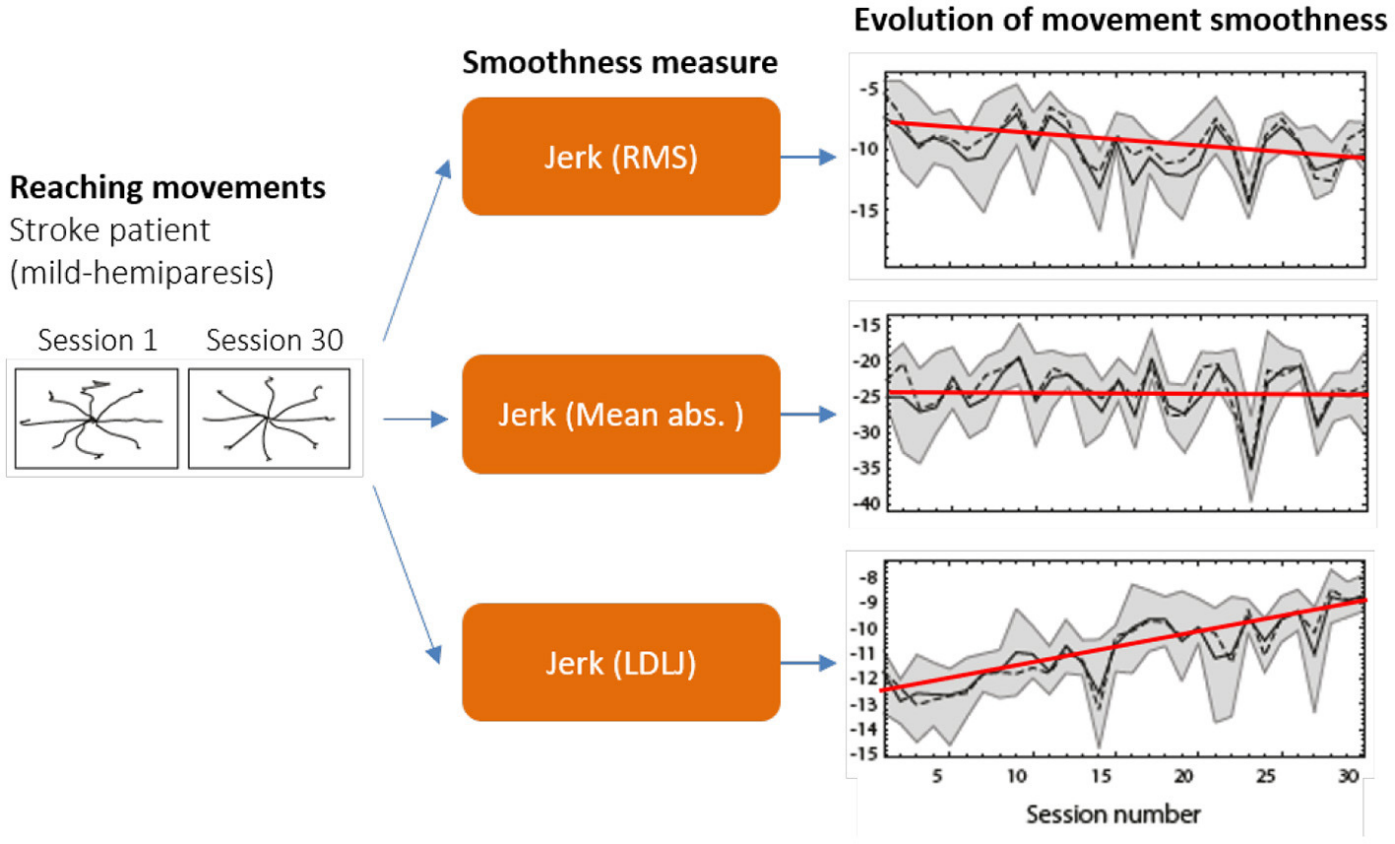
Different measures of smoothness can lead to vastly different conclusions. In this example, three jerk-based smoothness measures **(middle)** were applied to arm movements performed by a post-stroke subject in multiple directions **(left)**, over 30 training sessions **(right)**. The trends suggest three conflicting outcomes: Jerk (RMS) shows reduction in movement smoothness, indicating that the training was detrimental; Jerk (Mean abs.) indicates that the training had no effect; and Jerk (LDLJ) indicates that the training was beneficial. Data from Balasubramanian et al. (2012) with permission.

Quantitative measures of movement smoothness are of great interest as they allow us to evaluate the evolution of motor skill learning or recovery (Toosizadeh et al., 2015; Trehan et al., 2015; Moghaddas et al., 2019). Intuitively, movement smoothness is understood as a measure of non-intermittency, i.e., how uninterrupted a movement is, or how closely it resembles a movement with an initial period of acceleration followed by a period of deceleration (Hogan and Sternad, 2007, 2009; Balasubramanian et al., 2012, 2015). There have been several attempts to formally develop quantitative measures of movement smoothness (Rohrer and Hogan, 2006; Hogan and Sternad, 2007, 2009; Balasubramanian et al., 2012, 2015), but there is still no consensus on the most appropriate measure to use in different tasks or with different measurement technologies. This has led to the adoption of diverse measures that, when applied to the same data set, can generate contradicting results, leading to inconsistent and potentially incorrect interpretations (Figure 1).

To ensure appropriate quantification and interpretation of movement smoothness, valid, consistent, and reliable measures should be used. Previous work (Balasubramanian et al., 2012, 2015) has shown that, among several commonly used smoothness measures, only spectral arc length (SPARC) and log dimensionless jerk (LDLJ-V) possess these properties. Thus, SPARC and LDLJ-V have been used in multiple studies using data from different motion sensing technologies [e.g., image-based motion capture in Gulde and Hermsdörfer (2018), robotic devices in Colombo et al. (2017), IMUs in Rihar et al. (2014), or depth cameras in Abdi et al. (2016)]. In many cases, especially when movement velocity is not directly available, SPARC or LDLJ-V have been loosely adapted to other kinematic variables, e.g., acceleration from IMUs. Possible adaptations of these measures for different signals were tentatively proposed in Balasubramanian et al. (2015); however, they were not supported by any mathematical arguments or experimental validation. Thus, the properties of these adapted smoothness measures are unknown. Further, it is likely that the smoothness values derived from different variables (e.g., velocity or acceleration) are not directly comparable and may lead to different interpretations (e.g., Figure 1).

In this paper, we present a systematic investigation of the use of SPARC and LDLJ-V to evaluate movement smoothness using IMU data, i.e., linear acceleration from the accelerometer, and angular velocity from the gyroscope. For translational movements measured with IMUs (accelerometer), we show that SPARC cannot be used, and we propose a modification to the LDLJ-V to work with movement acceleration, LDLJ-A. For rotational movements, we show that SPARC can be directly applied to gyroscope data, while LDLJ-V is prone to errors. Using simulated and experimental data, we show conditions under which movement smoothness analysis can be carried out on both translational and rotational movements using an IMU. We also present an analysis of the nature of errors in movement smoothness analysis from IMU data. We strongly believe that the methods proposed in this paper are critical to (a) standardize analysis methods used in similar contexts (e.g., movement type, measurement technology, etc.); (b) avoid biased or inappropriate selection of smoothness measures; and (c) facilitate interpretation and direct comparison of results between studies.

## 2. Theoretical Background

To better understand the issues related to estimation of movement smoothness from IMU data, it is important to start with the construct of movement smoothness. Several groups have proposed that movement smoothness can be understood through the concept of submovements: “*…smooth movements are movements composed of a few submovements that are closely spaced in time, while unsmooth movements would result from the superposition of a larger number of submovements with loose temporal packing”* (Balasubramanian et al., 2012). Therefore, a valid and consistent movement smoothness measure must (Balasubramanian et al., 2012): (a) be dimensionless, i.e., independent of the movement amplitude and duration, and (b) have a monotonic response to change in the submovement characteristics in a movement.

With this in mind, we describe two main problems associated with the use of IMU data to estimate movement smoothness:

1. movement smoothness using SPARC and LDLJ-V requires knowledge of velocity; and
2. IMUs measure in their local reference frames, and reconstructing these data in an Earth-fixed reference frame is prone to errors that can greatly affect smoothness estimates.

### 2.1. Why SPARC and LDLJ Should Only Be Computed From Movement Velocity

Movement smoothness is a measure of movement quality, and intuitively, it should be an intrinsic property of a movement and not depend on the kinematic variables (position, velocity, acceleration, etc.) selected to quantify it. This means that given two movements *M*_*a*_ and *M*_*b*_, one could determine the same relative smoothness of these movements from different space representations, e.g., their velocities or accelerations. Although this idea is appealing on an intuitive level, mathematically it does not hold, because not all representations are equally informative about movement smoothness. Although this might seem obvious, several studies have estimated movement smoothness applying SPARC or LDLJ-V directly to acceleration signals, which is incorrect.

Movement smoothness is related to the concept of intermittency of the movement, which is also associated with the idea of temporal dispersion of submovements. One interpretation of movement intermittency is the presence of *movement arrest period* (MAP), which is a continuous interval of time within the overall movement duration where there is no movement, i.e., where all derivatives of position are uniformly zero. Movement velocity is the most direct indicator of movement intermittency caused by MAPs. This is because all time intervals with uniformly zero velocity are MAPs, while non-zero velocities indicate movement. This unique relationship, however, is lost with higher derivatives (acceleration, jerk, etc.), e.g., intervals with zero acceleration can either indicate an MAP or an interval with constant velocity.

SPARC uses submovements as a model of movement generation, resulting in the definition in Equation (1). This formulation quantifies dispersion of submovements in time, resulting in a monotonic response to the motion characteristics so that the smoothness measure decreases with the number of submovements and the inter-submovement interval (Balasubramanian et al., 2015). Because of its reliance on MAPs, and despite its dimensionless nature, SPARC applied to acceleration data looses its interpretability as a movement smoothness measure (see Appendix C). At the very best, one possible interpretation of SPARC applied to acceleration data from a movement *M* is that it represents the smoothness of a movement $\tilde{M}$ whose velocity profile is the same as movement *M*'s acceleration profile.

On the other hand, LDLJ-V does not rely directly on the concept of MAPs to quantify movement intermittency, but rather does this through the jerk term, i.e., changes to the movement acceleration. However, to make the measure dimensionless, the integrated squared jerk must be multiplied by a normalization factor. The definition of LDLJ-V in Equation (2) uses a normalization factor that depends on the peak of the speed profile *v*_*peak*_. Although this is not the only way to obtain a dimensionless measure, this specific normalization factor results in a measure that has a monotonic response to changes in the submovement characteristics of the underlying movement. If another normalization factor is used, this property, which is essential for the validity of the measure, might not necessarily hold.

These aspects are highly relevant for movement analyses where velocity cannot be reliably reconstructed, as in the case of IMUs.

**SPARC:**

(1) $$\begin{array}{l} \begin{array}{c} \lambda_{S}^{v}\left(\text{v}\right)≜-\int_{0}^{\omega_{c}}{\left[{\left(\frac{1}{\omega_{c}}\right)}^{2}+{\left(\frac{d\hat{V}\left(\omega \right)}{d\omega }\right)}^{2}\right]}^{\frac{1}{2}}d\omega ;\\ \hat{V}\left(\omega \right)=\frac{V\left(\omega \right)}{V\left(0\right)};\;V\left(\omega \right)=|F\left({\left\|\text{v}\left(t\right)\right\|}_{2}\right)| \end{array} \end{array}$$

**LDLJ based on velocity (LDLJ-V):**

(2) $$\begin{array}{l} \begin{array}{c} \lambda_{L}^{v}\left(\text{v}\right)≜-\ln\;\left(\frac{{\left(t_{2}-t_{1}\right)}^{3}}{v_{peak}^{2}}\int_{t_{1}}^{t_{2}}{\left\|\frac{d^{2}}{dt^{2}}\text{v}\left(t\right)\right\|}_{2}^{2}dt\right);\\ \;v_{peak}≜\underset{t\in \left[t_{1},t_{2}\right]}{\max}{\left\|\text{v}\left(t\right)\right\|}_{2}, \end{array} \end{array}$$

where **v**(*t*) represents the velocity (linear or angular) of a movement in the time domain, F(·) is the Fourier transform operator, ω_*c*_ is an adaptive cut-off frequency (see Balasubramanian et al., 2015 for details), and *t*_1_ and *t*_2_ are the movement start and stop times, respectively. In this paper, we assume that the models of movement generation used by SPARC and LDLJ-V to quantify translational movement smoothness are the same as their rotational counterparts—i.e., we can simply replace translational variables by the corresponding rotational ones.

### 2.2. The Kinematics Measured by an IMU

**Figure 2:**
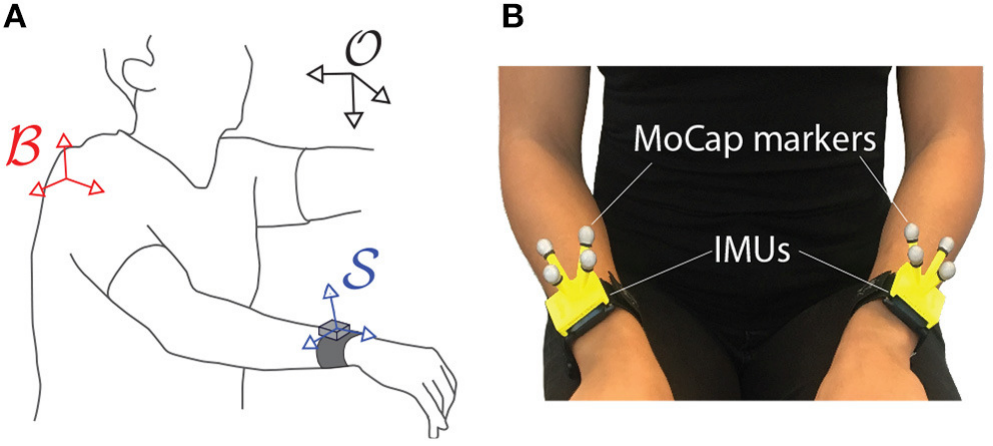
**(A)** Different coordinate frames of interest in movement analysis: inertial or earth-fixed O, body B, and sensor S reference frames. **(B)** Different technologies used to capture the kinematics of S: motion capture using reflective markers (MoCap) or Inertial Motion Units (IMUs). This setup was used to collect the data presented in section 4.

In human movement analysis, we are typically interested in translational and rotational motions of a single or a chain of body segments (e.g., arm or leg) relative to a body coordinate frame B (e.g., located at the shoulder or pelvis). Consider the scenario depicted in Figure 2A, where an IMU is placed at the wrist to measure the quality of discrete arm movements. Since movement smoothness should be computed from velocity, we are interested in the translational and rotational velocities of the wrist S, with respect to the shoulder reference frame B, i.e., ${\;}^{B}{\dot{\text{x}}}_{BS}$ and ${\;}^{B}\omega_{BS}$, respectively (refer to section Notatiion for details about conventions for the mathematical symbols). Note that both frames S and B move relative to an earth-fixed reference O.

An IMU typically includes a 3-axis accelerometer, which measures its linear acceleration ${\;}^{S}\text{a}\left(t\right)$, and a 3-axis gyroscope, which measures its angular velocity ${\;}^{S}\text{w}\left(t\right)$, expressed in the local (IMU sensor) reference frame S:

(3) $$\begin{array}{l} {\;}^{S}\text{a}\left(t\right){=}^{S}{\ddot{\text{x}}}_{OS}\left(t\right){+}^{S}\text{g}={\;}_{O}^{S}\text{R }\left(t\right)\left({\;}^{O}{\ddot{\text{x}}}_{OS}\left(t\right){+}^{O}\text{g}\right) \end{array}$$

(4) $$\begin{array}{l} {\;}^{S}\text{w}\left(t\right){=}^{S}\omega_{OS}\left(t\right)={\;}_{O}^{S}\text{R }{\left(t\right)}^{O}\omega_{OS}\left(t\right), \end{array}$$

where ${\;}^{O}\text{g}$ is the gravity vector, ${\;}^{O}{\text{x}}_{OS}\left(t\right)$ is the position of the IMU reference frame S with respect to O expressed in frame O, ${\;}_{O}^{S}\text{R }\left(t\right)$ is the rotation matrix representing O in S. For simplicity, we will drop the notation indicating the dependence on time *t* (e.g., ${\;}^{S}\text{a}\left(t\right)$ will be written as ${\;}^{S}\text{a}$). In the analysis presented here, we do not consider the contributions of measurement noise, as our primary goal is to demonstrate the effect of the physics of IMUs on smoothness analysis. Some work on the effect of noise on smoothness analysis can be found in Balasubramanian et al. (2012) and Balasubramanian et al. (2015).

Although important, the choice of reference frame when computing movement smoothness is rarely, if at all, discussed in existing literature. Theoretically, any reference frame could be used; however, to ensure consistency and the ability to compare results across studies, an Earth-fixed frame is a convenient choice as it is absolute to all researchers (at least those that are on Earth) and thus tends to be the reference frame of choice. Otherwise, comparisons would require accounting for the relative movement between the selected reference frames, which are typically not available.

It should be noted that B is physically attached to S through a set of rigid bodies interconnected through joints, and thus movements of B will be transmitted to S. Therefore, we can rewrite Equation (3) as (derivation in Appendix A):

(5) $$\begin{array}{l} {\;}^{S}\text{a}{=}^{S}\text{g}+{\;}_{B}^{S}\text{R }{\;}^{B}{\ddot{\text{x}}}_{BS}{+}^{S}{\ddot{\text{x}}}_{OB}+2{\;}_{O}^{S}\text{R }{\;}_{B}^{O}\dot{\text{R }}{\;}^{B}{\dot{\text{x}}}_{BS}+{\;}_{O}^{S}\text{R }{\;}_{O}^{S}\text{R }{\;}_{B}^{O}\ddot{\text{R }}{\;}^{B}{\text{x}}_{BS}, \end{array}$$

where the accelerometer readings from the IMU ${\;}^{S}\text{a}$ are composed of

- Linear acceleration due to gravity $\left({\;}^{S}\text{g}\right)$;
- Linear acceleration of S with respect to B $\left({\;}^{S}{\ddot{\text{x}}}_{BS}\right)$;
- Linear acceleration of B with respect to O (${\;}^{S}{\ddot{\text{x}}}_{OB}$);
- Coriolis acceleration of S due to rotation of frame B $\left(2{\;}_{O}^{S}\text{R }{\;}_{B}^{O}\dot{\text{R }}{\;}^{B}{\dot{\text{x}}}_{BS}\right)$; and
- Linear acceleration of S due to angular acceleration of frame B $\left({\;}_{O}^{S}\text{R }{\;}_{B}^{O}\ddot{\text{R }}{\;}^{B}{\text{x}}_{BS}\right)$;

Note that one could easily derive ${\;}^{B}{\dot{\text{x}}}_{BS}$ from position kinematics measured with optical motion capture systems. This, however, is not straightforward with IMU data.

Similarly, Equation (4) can be expressed as

(6) $$\begin{array}{l} {\;}^{S}\text{w}={\;}_{O}^{S}\text{R }{\;}_{B}^{O}\text{R }\left({\;}^{B}\omega_{BS}{+}^{B}\omega_{OB}\right){=}^{S}\omega_{BS}{+}^{S}\omega_{OB}, \end{array}$$

where the gyroscope data ${\;}^{S}\text{w}$ is composed of:

- Angular velocity of S with respect to B $\left({\;}^{S}\omega_{BS}\right)$,
- Angular velocity of B with respect to O $\left({\;}^{S}\omega_{OB}\right)$;

all expressed in the IMU sensor reference frame S.

Thus, in general, a single IMU cannot be directly used to analyze and interpret movements of isolated limbs without the knowledge of the translational and rotational kinematics of B with respect to O. This fact is perhaps obvious, but we think it is important to remark because this has been neglected in several previous studies. For example, there have been studies where researchers want to investigate arm movement control during activities of daily living, and IMUs are placed at the wrist but no information about the trunk is considered; or walking experiments where IMUs are placed at the ankles only. In such studies, reported movement smoothness results should be interpreted as the smoothness of the whole kinematic chain and body with respect to an Earth-fixed reference frame, and not just of the isolated limb on which the sensor is placed.

**The special case where the body reference frame is fixed**. In many experimental setups in movement neuroscience and neurorehabilitation, B is fixed with respect to O (e.g., the trunk is strapped to a chair). This ensures that the movement kinematics of the body parts of interest S with respect to B are related to the kinematics of S with respect to O through fixed affine transformation for position and linear transformation for its derivatives. If B is fixed with respect to O (i.e., ${\;}^{S}{\text{x}}_{OB}$ and ${\;}_{B}^{O}\text{R }$ are constant), ${\;}^{S}\text{a}$ reduces to

(7) $$\begin{array}{l} {\;}^{S}\text{a}={\;}_{B}^{S}\text{R }{\;}^{B}{\ddot{\text{x}}}_{BS}{+}^{S}\text{g} \end{array}$$

(8) $$\begin{array}{l} \Rightarrow^{B}{\dot{\text{x}}}_{BS}=\int_{t_{1}}^{t}{\;}_{S}^{B}\text{R }\left({\;}^{S}\text{a}{-}^{S}\text{g}\right)dt+\text{c}={\;}_{O}^{B}\text{R }{\;}^{O}{\dot{\text{x}}}_{OS}, \end{array}$$

where **c** is a constant. Considering only discrete movements, i.e., movements that are start and end with postures where all derivatives of position are zero, this implies that **c** = **0**.

Then, the *movement smoothness* of the translational motion of S, when B is fixed, is

(9) $$\begin{array}{l} \lambda^{v}\left({\;}^{B}{\dot{\text{x}}}_{BS}\right)=\lambda^{v}\left({\;}_{O}^{B}\text{R }{\;}^{O}{\dot{\text{x}}}_{OS}\right)=\lambda^{v}\left({\;}^{O}{\dot{\text{x}}}_{OS}\right) \end{array}$$

for both SPARC and LDLJ-V, since ${\;}_{O}^{B}\text{R }$ is fixed with respect to time.

For rotations, similar to the translational motion case, if B is fixed, i.e., ${\;}^{B}\omega_{OB}=\text{0}$, then,

(10) $$\begin{array}{l} {\;}^{B}\omega_{BS}={\;}_{S}^{B}\text{R }{\;}^{S}\text{w}={\;}_{O}^{B}\text{R }{\;}^{O}\omega_{OS}. \end{array}$$

Using the assumption that the smoothness of rotational movements can be analyzed in the same way as translational movements, Equation (10) allows us to estimate movement smoothness, when B is fixed, as

(11) $$\begin{array}{l} \lambda^{v}\left({\;}^{B}\omega_{BS}\right)=\lambda^{v}\left({\;}_{O}^{B}\text{R }{\;}^{O}\omega_{OS}\right)=\lambda^{v}\left({\;}^{O}\omega_{OS}\right). \end{array}$$

In other words, movement smoothness is invariant under fixed translation and rotation of reference frames.

### 2.3. Smoothness of Discrete Movements From IMUs

**Figure 3:**
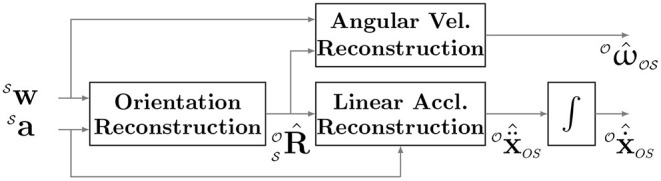
Reconstruction of kinematic variables of interest for estimating smoothness from IMU data.

In theory, and in the absence of noise, the most straightforward approach to estimate movement smoothness from IMU data would be to reconstruct translational and angular velocities with respect to O (${\;}^{O}{\hat{\dot{\text{x}}}}_{OS}$ and ${\;}^{O}{\hat{\omega }}_{OS}$) from the IMU measurements (Figure 3; Equations 3 and 4), then use SPARC or LDLJ-V to estimate smoothness. This This requires one to first estimate the orientation of the IMU during the course of the observed movement, i.e., compute ${\;}_{S}^{O}\hat{\text{R }}$, which is an estimate of the orientation of the IMU. However, in practice, errors in orientation reconstruction of S with respect to O result in errors in ${\;}^{O}{\hat{\dot{\text{x}}}}_{OS}$ and ${\;}^{O}{\hat{\omega }}_{OS}$, thus leading to inaccurate estimates of movement smoothness. Here, we analyze these issues for translational and rotational movements, and propose alternative ways to compute movement smoothness from IMU data. We will only consider **discrete movements** in our analysis, i.e., movements that start and end with a posture (Hogan and Sternad, 2007), such that,

(12) $$\begin{array}{l} {\;}^{O}{\dot{\text{x}}}_{OS}\left(t_{1}\right){=}^{O}{\dot{\text{x}}}_{OS}\left(t_{2}\right)=\text{0}\;\Rightarrow \;\int_{t_{1}}^{t_{2}}{\;}^{O}{\ddot{\text{x}}}_{OS}\left(t\right)dt=\text{0}. \end{array}$$

There are multitude ways orientation of an IMU can be reconstructed by combining acceleration, gyroscope, and magnetometer data (see Kok et al., 2017 for a review). Widely used methods include reconstruction by means of Kalman filtering (Luinge and Veltink, 2005; Sabatini, 2011) or complementary filters (Mahony et al., 2008; Madgwick et al., 2011).

#### 2.3.1. Translational Motion

To analyze the effects of kinematic reconstruction errors, the reconstructed linear acceleration from IMU data can be expressed as:

(13) $$\begin{array}{l} {\;}^{O}{\hat{\ddot{\text{x}}}}_{OS}={\;}_{S}^{O}\hat{\text{R }}{\;}^{S}\text{a}{-}^{O}\text{g} \end{array}$$

Let $\delta \text{R }={\;}_{S}^{O}\hat{\text{R }}{\;}_{O}^{S}\text{R }$ be the orientation reconstruction error; when orientation reconstruction is perfect, δ**R** = **I**. Equation (13) can be written as:

(14) $$\begin{array}{l} \begin{array}{l} {\;}^{O}{\hat{\ddot{\text{x}}}}_{OS}{={\;}_{S}^{O}\hat{\text{R }}}_{O}^{S}\text{R }({\;}^{O}{\ddot{\text{x}}}_{OS}{+}^{O}\text{g}){-}^{O}\text{g}\\ \;=\delta {\text{R }}^{O}{\ddot{\text{x}}}_{OS}+{\left(\delta \text{R }-\text{I}\right)}^{O}\text{g}\\ \;={\;}^{O}{\ddot{\text{x}}}_{OS}+\left(\delta \text{R }-\text{I}\right)({\;}^{O}{\ddot{\text{x}}}_{OS}{+}^{O}\text{g})\\ \;{=}^{O}{\ddot{\text{x}}}_{OS}+\delta \text{a}, \end{array} \end{array}$$

(15) $$\begin{array}{l} \begin{array}{l} {\;}^{O}{\hat{\dot{\text{x}}}}_{OS}=\int_{t_{1}}^{t}{\;}^{O}{\hat{\ddot{\text{x}}}}_{OS}dt{=}^{O}{\dot{\text{x}}}_{OS}+\int_{t_{1}}^{t}\delta \text{a}dt\\ \;={\;}^{O}{\dot{\text{x}}}_{OS}+\delta \text{v}, \end{array} \end{array}$$

where δ**a**, δ**v** are the linear acceleration and velocity reconstruction errors, respectively.

Thus, in the case of translational motion, using SPARC $\left(\lambda_{S}^{v}\right)$ or LDLJ-V $\left(\lambda_{L}^{v}\right)$ is problematic because of drift in ${\;}^{O}{\hat{\dot{\text{x}}}}_{OS}$ caused by integration of δ**a** and noise in the sensor data (which is not considered in these equations) (Thong et al., 2004; Kok et al., 2017; Picerno, 2017b; Alvarez et al., 2018). In human movement analysis, the magnitude of gravity ${\;}^{O}\text{g}$ is much larger than linear accelerations of typical arm movements, which can result in significant drift in ${\;}^{O}{\hat{\dot{\text{x}}}}_{OS}$ due to reconstruction errors; this problem is exacerbated in the case of slow, long-duration movements, as seen in patients with movement impairments.

##### 2.3.1.1. Proposed solution: LDLJ-A for acceleration data

As discussed in section 2.1, applying SPARC or LDLJ-V to acceleration signals does not provide the smoothness of the movement represented by these acceleration signals. It is not clear how SPARC could be calculated from acceleration data, given that there is no simple relationship between the Fourier magnitude spectra of velocity and acceleration signals. However, we can obtain jerk from acceleration, and choose an appropriate normalization factor to allow the LDLJ-V to work directly with acceleration data, which results in the new measure (LDLJ-A) applied to acceleration (see Appendix B for details about its properties). This measure avoids integration of the reconstructed acceleration signal, and thus the errors associated with drift in the reconstructed velocity.

**LDLJ based on acceleration (LDLJ-A):**

(16) $$\begin{array}{l} \lambda_{L}^{a}{(}^{O}{\overset{..}{x}}_{OS})≜-\ln\;(\frac{t_{2}-t_{1}}{a_{peak}^{2}}I_{j});\\ \;I_{j}≜\int_{t_{1}}^{t_{2}}{\left\|{\;}^{O}{\overset{...}{x}}_{OS}(t)\right\|}_{2}^{2}dt;\\ \;a_{peak}≜\underset{t\in [t_{1},t_{2}]}{\max}{\left\|{\;}^{O}{\bar{\overset{..}{x}}}_{OS}(t)\right\|}_{2};\\ \;{\;}^{O}{\bar{\overset{..}{x}}}_{OS}(t){≜}^{O}{\overset{..}{x}}_{OS}(t)-\frac{1}{t_{2}-t_{1}}\int_{t_{1}}^{t_{2}}{\;}^{O}{\overset{..}{x}}_{OS}(t)dt \end{array}$$

where ${\;}^{O}{\bar{\ddot{\text{x}}}}_{OS}$ is the mean subtracted acceleration. For discrete movements, ${\;}^{O}{\bar{\ddot{\text{x}}}}_{OS}{=}^{O}{\ddot{\text{x}}}_{OS}$ (Equation 12). However, for reconstructed acceleration data ${\;}^{O}{\hat{\ddot{\text{x}}}}_{OS}$ from discrete movements, the mean might not be zero due to imperfect removal of gravity, and data segmentation practices to determine movement onset and termination. Removing the mean reduces the amount of overestimation in the acceleration peak of ${\;}^{O}{\hat{\ddot{\text{x}}}}_{OS}$. It should be noted that the mean value of ${\;}^{O}{\hat{\ddot{\text{x}}}}_{OS}$ does not affect the jerk integral term *I*_**j**_.

#### 2.3.2. Rotational Motion

Dealing with rotational motion data from an IMU is easier, since: (a) the sensor directly measures angular velocity via the gyroscope, albeit in the local sensor reference frame, and (b) the signal is not affected by gravity.

For SPARC, the sensor orientation is irrelevant, since the operator ·_2_ is rotation invariant. The rotational speed can be computed directly from the gyroscope data, i.e., ${\left\|{\;}^{O}\omega_{OS}\right\|}_{2}=\;{\left\|{\;}_{S}^{O}R{\;}^{O}\omega_{OS}\right\|}_{2}=\;{\left\|{\;}^{S}w\right\|}_{2}$. Therefore, $\lambda_{S}^{v}\left({\;}^{O}\omega_{OS}\right)=\lambda_{S}^{v}\left({\;}^{S}\text{w}\right)$.

On the other hand, LDLJ-V requires ${\;}^{O}{\hat{\omega }}_{OS}$, which is affected by the orientation reconstruction error:

(17) $$\begin{array}{l} \begin{array}{l} {\;}^{O}{\hat{\omega }}_{OS}{=}_{S}^{O}{\hat{\text{R }}}^{S}\text{w}=\delta {\text{R }}^{O}\omega_{OS}\\ \;{=}^{O}\omega_{OS}+{\left(\delta \text{R }-\text{I}\right)}^{O}\omega_{OS}\\ \;{=}^{O}\omega_{OS}+\delta \omega , \end{array} \end{array}$$

where δ**ω** is the rotational velocity reconstruction error. Since the rotational jerk is estimated by double differentiation, it is important to understand how errors in orientation reconstruction affect the computation of LDLJ-V from gyroscope data.

We note that there is an alternative formulation to compute the magnitude of rotational jerk (and thus LDLJ-V) that is unaffected by the sensor rotation (see Appendix D for details). However, this formulation was found to be sensitive to practical implementation issues (e.g., choice of numerical differentiation methods, sampling frequency, etc.) and thus, needs further investigation before general recommendations can be made for its use in movement smoothness analysis.

In the next sections, we analyze the effect of reconstruction errors on the smoothness estimates of translational and rotational movements using simulated (section 3) and experimental (section 4). For experimental data, we also analyze the agreement between smoothness measures calculated from camera-based motion capture and IMU data.

## 3. Validation Using Simulated Data

### 3.1. Simulation Data: Methods

We simulated discrete point-to-point movements to test the proposed methods with knowledge of the ‘ground truth’ and full control over reconstruction errors. To cover a wide range of movements that are relevant for human movement analysis, 1*s* duration, 15*cm* length minimum jerk trajectories with varying number of via-points were generated. The following parameters were used:

(i) Number of via-points: the number of via-points (spatio-temporal constraints) can affect movement smoothness. We used a subset of simulated discrete point-to-point movements from the analysis presented in Appendix B; twenty-five different trials with different number of via-points (*N*_*via*_ = {1, 2, 5, 10}) were selected for this analysis.

(ii) Movement duration: for a fixed movement amplitude, movement duration controls the magnitude of the acceleration and velocity. Acceleration of shorter duration movements are dominated by the linear acceleration component ${\;}^{S}{\ddot{\text{x}}}_{OS}$, while longer duration movements are dominated by the gravity component ${\;}^{S}\text{g}$. Each of the 1*s* duration simulated movements were time-scaled to *T* = {2.5, 5, 10, 20}*s*. The corresponding velocity and acceleration were scaled as

$$\begin{array}{l} {\text{v}}_{{\;}_{T}}=\frac{{\text{v}}_{1}}{T}\;{\text{a}}_{{\;}_{T}}=\frac{{\text{a}}_{1}}{T^{2}} \end{array}$$

where **v**_1_ and **a**_1_ are the velocity and acceleration of the 1*s* duration movement.

(iii) Orientation reconstruction error: orientation reconstruction errors were simulated as stochastic time-series of rotation matrices, using the following parameterization,

(18) $$\begin{array}{l} \delta \text{R}\left(t\right)={\text{R}}_{z}\left(\alpha \left(t\right)\right){\text{ R}}_{y}\left(\beta \left(t\right)\right){\text{ R}}_{x}\left(\gamma \left(t\right)\right), \end{array}$$

where **R**_*x*_(·), **R**_*y*_(·), and **R**_*z*_(·) are the elementary rotation matrices about the *x*, *y*, and *z* axes, respectively; α(*t*), β(*t*), and γ(*t*) are Euler angles that determine the amount of rotation about each axis. The time-series of these Euler angles were realizations of Gaussian Brownian noise (integration of white noise), which were low-pass filtered through a moving average filter (window size of 0.5*s*). The angles were scaled such that $\max_{t}\alpha \left(t\right)=\max_{t}\beta \left(t\right)=\max_{t}\gamma \left(t\right)=\theta_{max}$, $\theta_{max}\in \left\{5{}^{\circ},25{}^{\circ},50{}^{\circ}\right\}$. θ_*max*_ determines the maximum amount of orientation reconstruction error represented by δ**R**. For each movement trial with a fixed number of via points and duration, we simulated five different realizations of the Euler angles for each value of θ_*max*_.

The simulated movements and orientation reconstruction errors were used to generate the reconstructed linear acceleration and angular velocity data as (from Equations 15 to 17)

$$\begin{array}{c} {\;}^{O}{\hat{\ddot{\text{x}}}}_{OS}=\delta {\text{R }}^{O}{\ddot{\text{x}}}_{OS}+{\left(\delta \text{R }-\text{I}\right)}^{O}\text{g}\\ {\;}^{O}{\hat{\omega }}_{OS}=\delta {\text{R }}^{O}\omega_{OS}=\delta {\text{R }}^{O}{\dot{\text{x}}}_{OS} \end{array}$$

where ${\;}^{O}{\dot{\text{x}}}_{OS}$ and ${\;}^{O}{\ddot{\text{x}}}_{OS}$ are the simulated minimum jerk movements, and δ**R** is the simulated sensor orientation reconstruction error (Equation 18). For the gyroscope data, we simply treated the simulated linear movements as rotational movements.

The values for the different factors used in the simulation analysis (Table 1) resulted in a total of 6,000 movements (= 4 number of via points × 25 repetitions × 4 movement durations × 3 orientation errors × 5 repetitions).

**Table 1:** Summary of parameters used to analyze the effect of reconstruction error on movement smoothness.

**Parameter**	**Values**
No. of via points	{1, 2, 5, 10} (25 reps)
Duration (*s*)	{2.5, 5, 10, 20}
Reconstruction error	{5°, 25°, 50°} (5 realizations)

*A total of 6,000 simulated movements were generated*.

Movement smoothness of the simulated movements using the reconstructed linear acceleration and angular velocity were estimated by:

- Applying $\lambda_{L}^{a}$ to the original movement acceleration ${\;}^{O}{\ddot{\text{x}}}_{OS}$ and the reconstructed acceleration ${\;}^{O}{\hat{\ddot{\text{x}}}}_{OS}$.
- Applying $\lambda_{S}^{v}$ and $\lambda_{L}^{v}$ to the original angular velocity ${\;}^{O}\omega_{OS}$ and reconstructed angular velocity ${\;}^{O}{\hat{\omega }}_{OS}$.

### 3.2. Simulation Data: Analysis

To better understand how the reconstruction error affects smoothness estimates, we quantified the following properties of δ**R**:

- First derivative: From Equation (18)
  (19) $$\begin{array}{l} \dot{\delta \text{R}}=\frac{d}{dt}\left(\delta \text{R}\right)=\dot{\alpha }\frac{\partial {\text{R}}_{z}}{\partial \alpha }{\text{R}}_{y}{\text{R}}_{x}+\dot{\beta }{\text{R}}_{z}\frac{\partial {\text{R}}_{y}}{\partial \beta }{\text{R}}_{x}\\ \;+\dot{\gamma }{\text{R}}_{z}{\text{R}}_{y}\frac{\partial {\text{R}}_{x}}{\partial \gamma }\Rightarrow {\left\|\dot{\delta \text{R}}\right\|}_{2}\propto \theta_{max} \end{array}$$
- Dynamic range, defined as
  (20) $$\begin{array}{l} \Delta_{\delta \text{R }}≜\underset{t_{i},t_{j}\in \left[t_{1},t_{2}\right]}{\max}{\left\|\delta \text{R }{\left(t_{i}\right)}^{⊤}\delta \text{R }\left(t_{j}\right)-\text{I}\right\|}_{2}, \end{array}$$

which provides a measure of the largest mismatch between two rotation matrices in the time series δ**R**(*t*).

It should be noted that the magnitudes of ${\left\|\dot{\delta \text{R }}\right\|}_{2}$ and Δ_δ**R**_ are unaffected by fixed offsets in the orientation reconstruction error. $\dot{\delta \text{R }}$ and Δ_δ**R**_ play an important role in determining how the nature of orientation reconstruction errors affect movement smoothness estimated using LDLJ-A (refer to Appendix E).

In the case of translational movements, the relative magnitude of the true linear acceleration and gravity is useful to gauge the relevance of the sensor measurement to quantify the movement in question. A rough estimate that can be obtained from accelerometer data is the sensor-to-gravity ratio (SGR):

(21) $$\begin{array}{l} \text{SGR}=\frac{1}{{\left\|{\;}^{O}\text{g}\right\|}_{2}}\sqrt{\frac{1}{t_{2}-t_{1}}\int_{t_{1}}^{t_{2}}{\left\|{\;}^{S}\text{a}\right\|}_{2}^{2}dt}. \end{array}$$

SGR can only assume non-negative real values. An accelerometer that is free-falling with respect to an Earth-fixed reference frame will measure **0** acceleration, and thus *SGR* = 0. In general, increasing values of SGR greater than 1 indicate an increasing contribution from the movement's linear acceleration.

We also evaluated the effect of orientation reconstruction error on the magnitudes of jerk and mean-subtracted acceleration. Effects were further quantified in terms of the percentage relative error in the jerk integral and acceleration peak terms:

(22) $$\begin{array}{l} \text{\% Jerk Error}≜100\times \left(\frac{{Î}_{\text{j}}-I_{\text{j}}}{I_{\text{j}}}\right)\;\text{and}\\ \text{\% Accl. Peak Error}≜100\times \left(\frac{{â}_{peak}-a_{peak}}{a_{peak}}\right) \end{array}$$

where Î_**j**_ is the integral of the squared magnitude of the reconstructed linear acceleration.

Smoothness estimates from the original and reconstructed data were compared using Pearson correlation coefficients. Further, the relative error ϵ between the smoothness of the original and reconstructed movements was calculated as:

(23) $$\begin{array}{l} \epsilon =\frac{\lambda \left(\hat{\text{y}}\right)-\lambda \left(\text{y}\right)}{\left|\lambda \left(\text{y}\right)\right|}, \end{array}$$

where λ(·) is the smoothness measure, **y** and $\hat{\text{y}}$ are the original and reconstructed movement kinematic variables of interest, respectively.

Finally, we analyzed how the correlation and relative error ϵ varied as a function of SGR. This was done by first selecting all movements with SGR greater than or equal to a threshold, and then estimating the correlation coefficient and the relative error ϵ for this set of movements; these plots were generated for SGR thresholds ∈ [1.0, 1.05, 1.1, 1.2, 1.5, 1.75, 2.0, 2.5].

All simulated data and Python code for their analysis presented in this paper is available here.

### 3.3. Simulated Data: Results and Discussion

**Figure 4:**
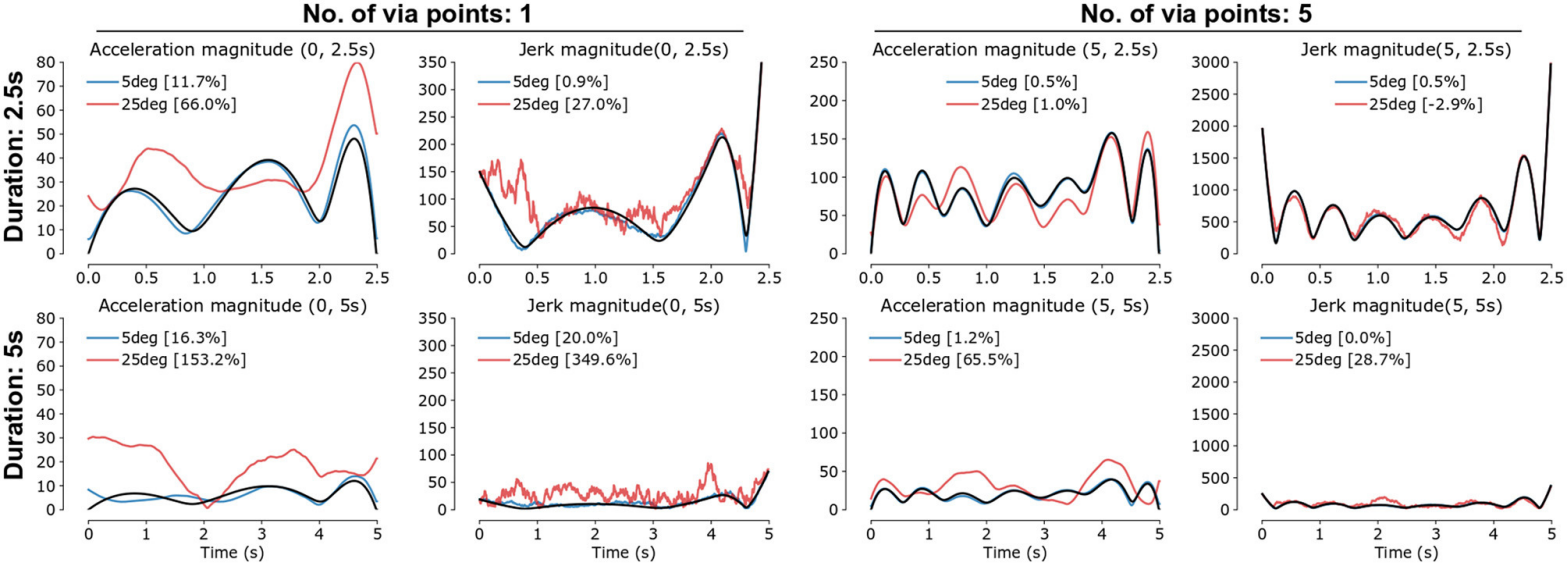
Effect of orientation reconstruction error on the magnitudes of reconstructed mean-subtracted acceleration $\left(\|{\;}^{O}{\ddot{\text{x}}}_{OS}{-}^{O}{\bar{\ddot{\text{x}}}}_{OS}{\|}_{2}\right)$ and jerk $\left(\|{\;}^{O}{\overset{.}{\text{x}}}_{OS}{\|}_{2}\right)$ for simulated data with zero (black), 5° (blue) and 25° (red) orientation error magnitudes. Sample simulated movements of 2.5*s* (top row) or 5*s* (bottom row) duration, with 1 (two columns on left) or 5 (two columns on right) via points. The percent relative error in the jerk integral and peak acceleration terms are in the legends of each subplot.

Orientation reconstruction error δ**R** has varying effects on the reconstructed acceleration and jerk (Figure 4). Movements with larger acceleration and jerk magnitudes had lower relative errors in jerk integral and peak acceleration. The relative errors for longer duration movements were higher than for shorter duration movements; this is due to increased relative contribution from the gravity term compared to the linear acceleration in longer duration movements (refer to Appendix E for details). Furthermore, for a given movement duration, movements with larger number of via points tend to have less relative errors due to their larger acceleration and jerk. We demonstrate in Appendix E that errors in the jerk integral and acceleration peak are determined by the derivative $\dot{\delta \text{R }}$ and the dynamic range Δ_δ**R**_ of the orientation reconstruction errors, respectively, rather than the magnitude of the orientation reconstruction error ‖δ**R** − **I**‖_2_.

**Figure 5:**
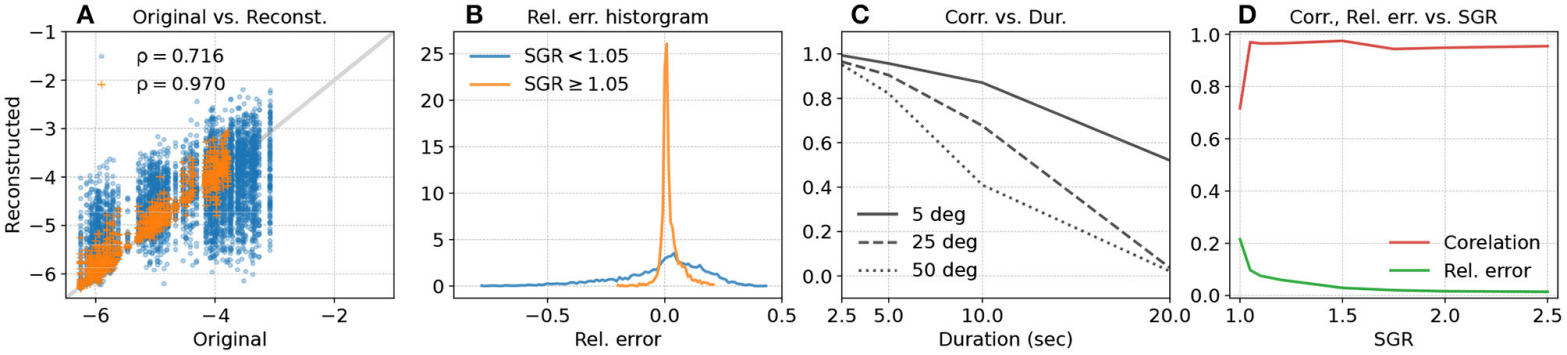
Simulation-based evaluation of LDLJ-A as smoothness estimate for translational movements. **(A)** Smoothness of reconstructed movements as a function of smoothness of the corresponding original movement for all simulated movements (blue), and movements with *SGR* ≥ 1.05 (orange), with corresponding Pearson correlation coefficients ρ. **(B)** Histogram of the relative error between smoothness of the original and the reconstructed movements for *SGR* < 1.05 (blue) and *SGR* ≥ 1.05 (orange). **(C)** Correlation between smoothness of original and reconstructed movements as a function of movement duration for different orientation reconstruction errors. **(D)** Correlation (red) and relative error (green) between smoothness from original and reconstructed movement as a function of SGR.

Orientation reconstruction errors also affect smoothness estimates of translational movements (Figure 5):

- Movements with fewer submovements (i.e., smoother movements) tend to have larger errors in their smoothness estimates. This is seen in the larger spread of points about the *y* = *x* line for movements with high original smoothness values (Figure 5A). This is because, in the simulated movements, for a given movement duration movements with fewer number of via points have lower acceleration and jerk magnitudes.
- Movements with larger linear acceleration relative to gravity (*SGR* ≥ 1.05) have smoothness values that are better correlated (Figure 5A) to that of the original movement, and have smaller reconstruction errors (Figure 5B). Increasing SGR results in better agreement between the smoothness values of the original and reconstructed movements (Figure 5D).
- Larger orientation reconstruction errors (θ_*max*_) resulted in poorer correlation between the true and reconstructed smoothness values (Figure 5C). For a given amount of orientation reconstruction error, the correlation decreases with increasing movement duration due to reduction in the acceleration and jerk magnitudes.

**Figure 6:**
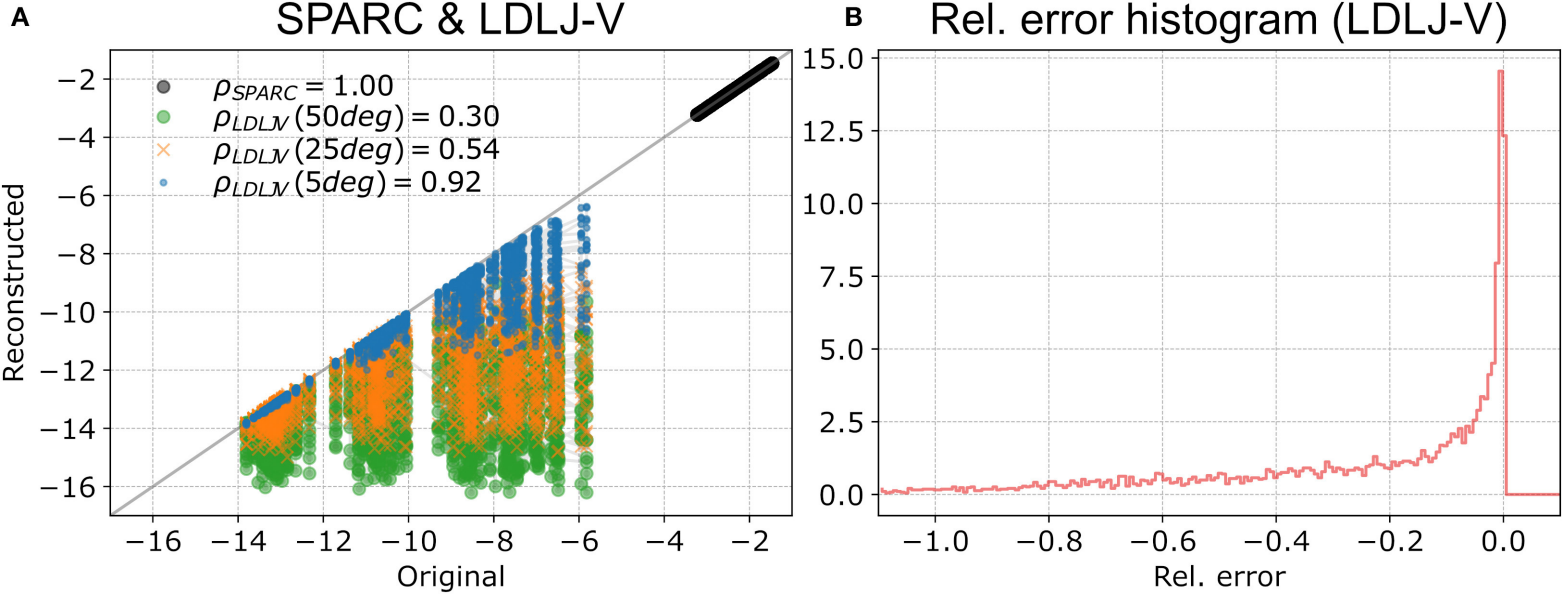
Evaluation of $\lambda_{S}^{v}$ and $\lambda_{L}^{v}$ to estimate smoothness of simulated rotational movements. **(A)** Smoothness of original $\lambda_{*}^{v}\left({\;}^{O}\omega_{OS}\right)$ vs. reconstructed movements $\lambda_{*}^{v}\left({\;}^{O}{\hat{\omega }}_{OS}\right)$ for SPARC $\lambda_{S}^{v}$ (black circles) and LDLJ-V $\lambda_{L}^{v}$ with maximum orientation reconstruction errors of 5deg (blue), 25deg (orange), and 50deg (green). The Pearson correlation coefficients are in the legend. **(B)** Histogram of the relative error between the $\lambda_{L}^{v}$ smoothness of the original and reconstructed movements.

For rotational movements (Figure 6), as expected, there was perfect correlation between smoothness of the original and reconstructed movements for SPARC regardless of reconstruction error (ρ = 1). However, LDLJ-V had a much lower correlation of ρ = 0.51 overall due to the orientation reconstruction errors. As expected, the correlation increased with decreasing orientation reconstruction error (Figure 6A). Additionally, correlation was poorer for smoother movements. The error in smoothness is larger for smoother movements, reaching up to 100% relative error (Figure 6B), due to the error in jerk calculation from ${\;}^{O}{\hat{\omega }}_{OS}$. In this case, the errors in smoothness estimate are entirely due to the jerk integral term, which depends on both $\dot{\delta \text{R }}$ and $\ddot{\delta \text{R }}$. The long tail of the histogram of the errors implies that there can large errors in smoothness estimated using LDLJ-V.

## 4. Validation Using Human Movement Data

### 4.1. Human Movement Data: Methods

To evaluate the consistency of estimating movement smoothness in practice, we applied the SPARC, LDLJ-V, and LDLJ-A to human arm movement data collected simultaneously with an optical passive marker-based motion capture system (MoCap) and IMUs. We used a set of 250 upper-limb movements, collected from 4 post-stroke individuals with different levels of arm impairment. Data were not collected for the specific purpose of this paper; they were collected during the patients' stay at the cereneo center for Neurology and Rehabilitation (Vitznau, Switzerland) as part of a different research study (BASEC ID 2017-00199). We only used anonymized data from patients who authorized ‘further use of data’. The analysis presented here does not fall into the category of Human Research, according to the Human Research Act (Art. 2) defined by the Swiss Federal Council; thus, no further ethical approval was required.

The kinematic dataset consisted of patients performing all tasks of the ARAT (Arm Research Action Test) with both their right and left arms. An optical motion capture system (Qualysis, Göteborg, Sweden) and one IMU worn on each wrist (ZurichMOVE, ETH Zurich, Switzerland) were used to track the kinematics of both arms and body. Motion capture is the process of recording movements of objects in 3D space (Winter, 2009). In our case, reflective markers were placed on the body and recorded by cameras that emit infrared light. The images from multiple cameras are used to reconstruct the body's movement on the computer (we refer the interested reader to Colyer et al. (2018) for a historic perspective and further details). To track the movement simultaneously with the two systems, a cluster of passive-reflective markers was attached to each of the IMUs as shown in Figure 2B.

Marker data for each ARAT task were tracked and exported to Visual3D (C-Motion, Germantown MD, USA), then low-pass filtered with a zero-lag 2^*nd*^ order Butterworth filter with cut-off frequency at 20 Hz. A rigid-body model was used to calculate the position (${\;}^{O}{\text{x}}_{OS}$), angular velocity (${\;}^{O}{\hat{\omega }}_{OS}$) and orientation (${\;}_{S}^{O}{\hat{\text{R }}}_{\text{MoCap}}$) of each cluster of markers representing the IMUs.

Data from the IMUs were a collected as continuous data streams, and the MoCap data were used to align the data in time and segment it into each ARAT task. Data from each IMU consisted of acceleration (${\;}^{S}\text{a}$), angular velocity (${\;}^{S}\text{w}$), and quaternions representing the orientation of the IMU, which were used to compute the orientation of the sensor ${\;}_{S}^{O}{\hat{\text{R }}}_{\text{IMU}}$.

Linear velocity was obtained by numerical differentiation of position data ${\;}^{O}{\text{x}}_{OS}$ (MoCap data) or integration of the rotated accelerometer signal minus gravity $\left({\;}_{S}^{O}{\hat{\text{R }}}_{\text{IMU}}{\;}^{S}\text{a}-{\;}^{O}\text{g}\right)$ (IMU data). To further understand the effect of changes in orientation in smoothness estimates, we added ‘noise’ to the reconstructed orientation of the sensor ${\;}_{S}^{O}{\hat{\text{R }}}_{\text{IMU}}$. For each of the 250 upper-limb movements, 100 stochastic time-series rotation matrices (**R**_noise_) with varying Euler angles ranging from 5 deg to 50 deg were generated, similarly to how orientation reconstruction errors were generated in section 3. This resulted in 25,000 movements with varying orientation estimates given by ${\text{R }}_{\text{noise}}{\;}_{S}^{O}{\hat{\text{R }}}_{\text{IMU}}$.

Smoothness measures were computed for both IMU and MoCap data. For SPARC, the adaptive cut-off frequency was chosen to be less than or equal to 6 Hz (i.e., ω_*c*_ ≤ 12π in Equation (1)). Thus, for LDLJ-V, signals were low-pass filtered with a zero-lag 2^*nd*^ order Butterworth filter with a cut-off frequency at 6 Hz.

### 4.2. Human Movement Data: Analysis

The agreement between IMU and MoCap smoothness values was quantified using Bland-Altman analysis (Giavarina, 2015) as a percentage relative to the smoothness quantified from MoCap (relative error), as MoCap is the ‘gold standard’ technology for movement analysis. Limits of agreement are reported as 1.96 times the standard deviation of the computed values and are shown in the figures with a 95% confidence interval.

### 4.3. Human Movement Data: Results and Discussion

**Figure 7:**
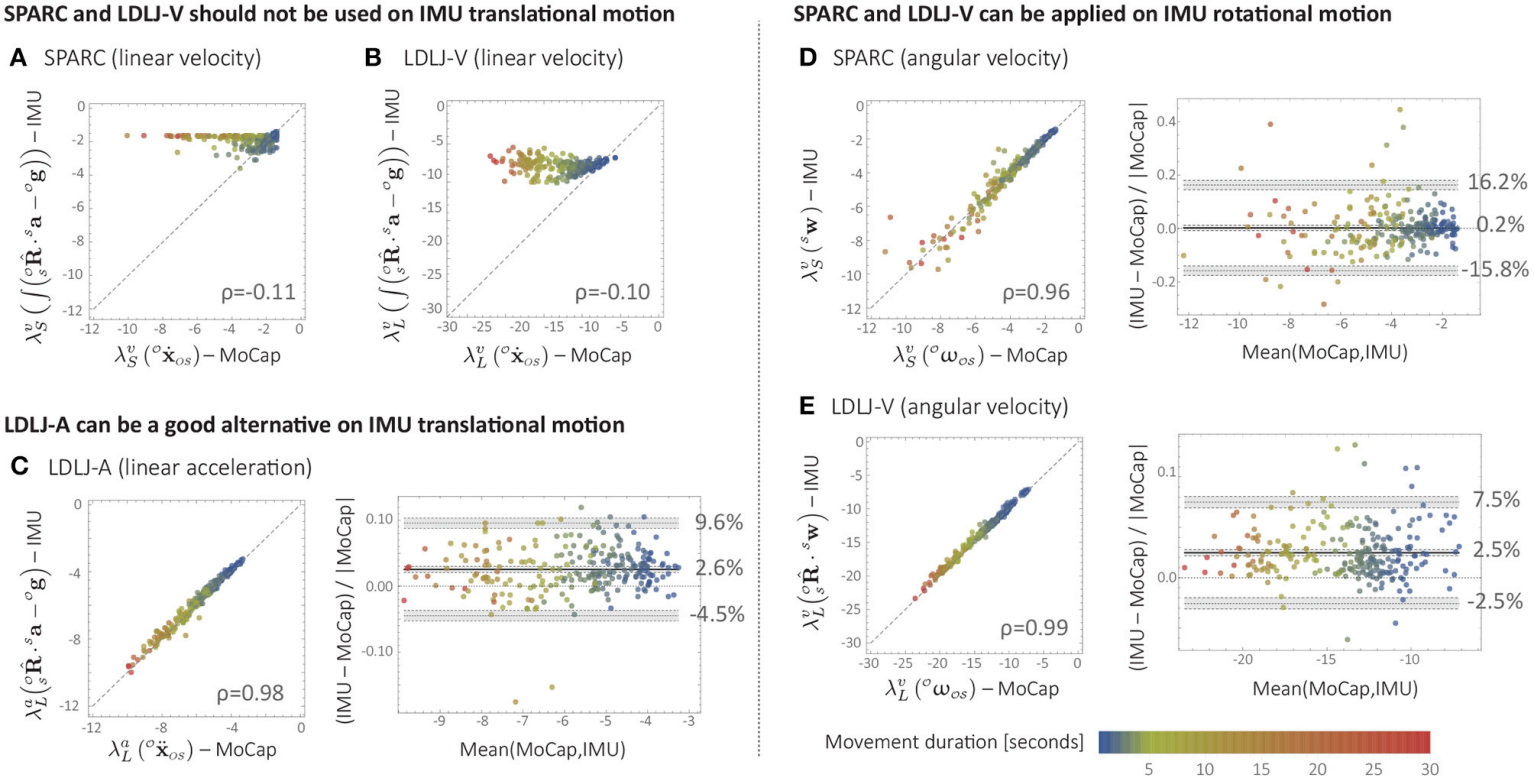
Comparison between smoothness estimates from Motion Capture (MoCap) and IMU data during upper-limb movements of stroke survivors. **(A)** SPARC (linear velocity). **(B)** LDLJ-V (linear velocity). **(C)** LDLJ-A (linear acceleration). **(D)** SPARC (angular velocity). **(E)** LDLJ-V (angular velocity).

For linear velocity, MoCap- and IMU-based smoothness estimates were highly uncorrelated for both SPARC (ρ = −0.11; Figure 7A) and LDLJ-V (ρ = −0.10; Figure 7B). As expected (see section 2.3.1), the drift caused by integration significantly affected the smoothness estimated from the IMU data. For SPARC, the drift causes a relative increase in the DC frequency component of the speed signal. This, in turn, decreases the adaptive cut-off frequency ω_*c*_ (Equation 1), resulting in a shorter spectral arc length, and thus a smoother estimate. For LDLJ-V, the drift results in the over-estimation of *v*_*peak*_, resulting in a smaller normalization factor, and thus a smoother estimate.

MoCap- and IMU-derived smoothness computed using LDLJ-A (Figure 7C) were highly correlated (ρ = 0.98). The limits of agreement indicate that IMU-derived estimates were smoother than those derived from MoCap, with a mean bias of 2.6% (range from −4.5 to 9.6%). One explanation for the bias could be the amplification of noise in the MoCap position data due to triple numerical differentiation needed for computing LDLJ-A, resulting in less smooth values. The overestimation of smoothness computed from IMU data was also seen with simulated movements in Figure 5A, where movements with smoothness values less than −4 are mostly on or above the identity line; the smoothness values of all movements in Figure 7C are also lower than −4.

**Figure 8:**
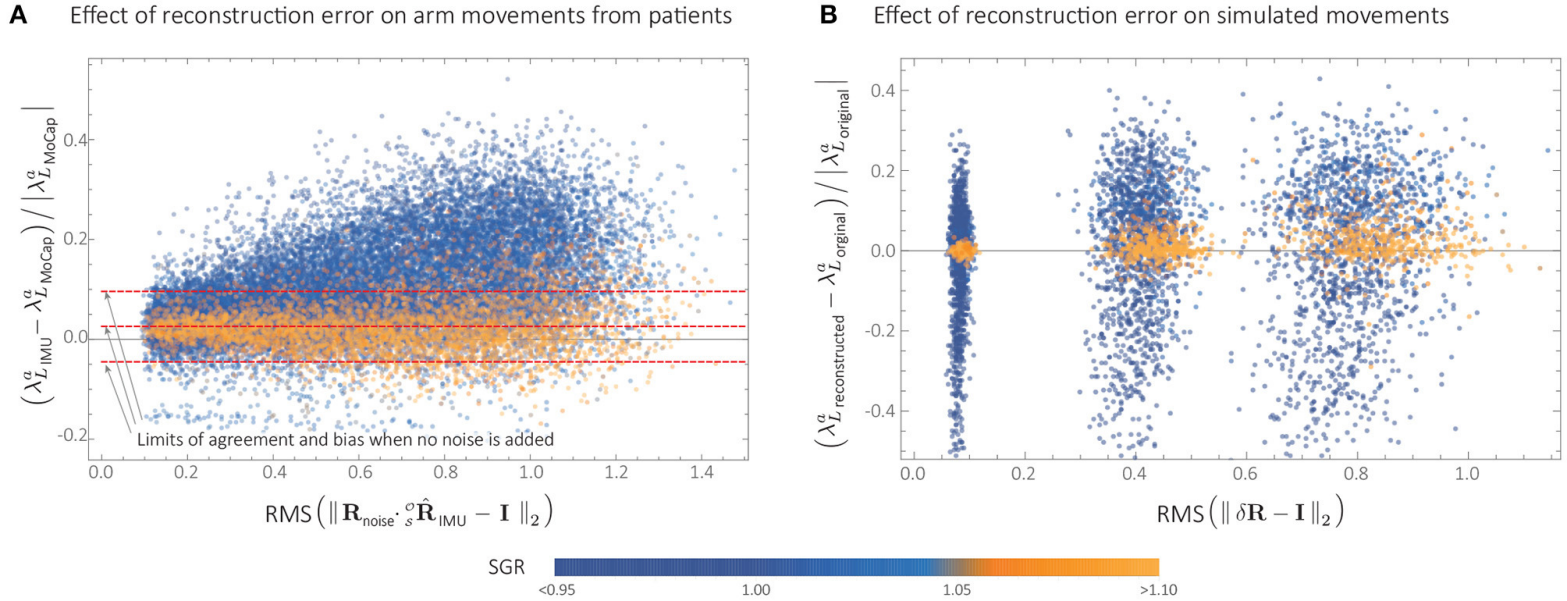
Effect of orientation reconstruction error on LDLJ-A for experimental and simulated data. **(A)** Agreement between MoCap and IMU from experimental data, when simulated noise (**R**_noise_) is added to the rotation matrix ${\;}_{S}^{O}{\hat{\text{R }}}_{\text{IMU}}$ obtained from patient data. **(B)** Relative error ϵ (defined in Equation 23) when simulated reconstruction error δ**R** is added to synthetic movement data. Here, the metric *RMS*(δ**R** − **I**_2_) is used as measure of orientation reconstruction error.

Despite the large number of experimental movements with *SGR* < 1.05, points in Figure 7C had little spread compared to Figure 5A, and the IMU- and MoCap-based smoothness were highly correlated. This was likely due to small reconstruction errors in ${\;}_{S}^{O}{\hat{\text{R }}}_{\text{IMU}}$ from this particular IMU sensor. Indeed, when simulated noise was added to ${\;}_{S}^{O}{\hat{\text{R }}}_{\text{IMU}}$ (see Figure 8A), the relative error between MoCap and IMU increased significantly.

For rotational movement, the MoCap- and IMU-derived smoothness values were highly correlated for both SPARC (ρ = 0.96; Figure 7D) and LDLJ-V (ρ = 0.99; Figure 7E). While SPARC resulted in almost no significant bias in relative error (0.2%), LDLJ-V had a relative error bias of 2.5%. On the other hand, the limits of agreement were smaller for LDLJ-V (−2.5 to 7.5%) compared to SPARC (−15.8 to 16.2%). The poorer levels of agreement of SPARC than of LDLJ-V was surprising, given that: (a) for angular velocity, SPARC is unaffected by rotation ${\;}_{S}^{O}{\hat{\text{R }}}_{\text{IMU}}$; and (b) SPARC has been shown to be more robust to noise than LDLJ-V (Balasubramanian et al., 2012, 2015). The high correlation between MoCap- and IMU-derived smoothness values for LDLJ-V agrees with the simulated data. These experimental rotational movements were relatively unsmooth, which tend to have better agreement with the true smoothness values for relatively small reconstruction errors (Figure 6). This is further discussed in section 5.2.

## 5. Discussion

**Figure 9:**
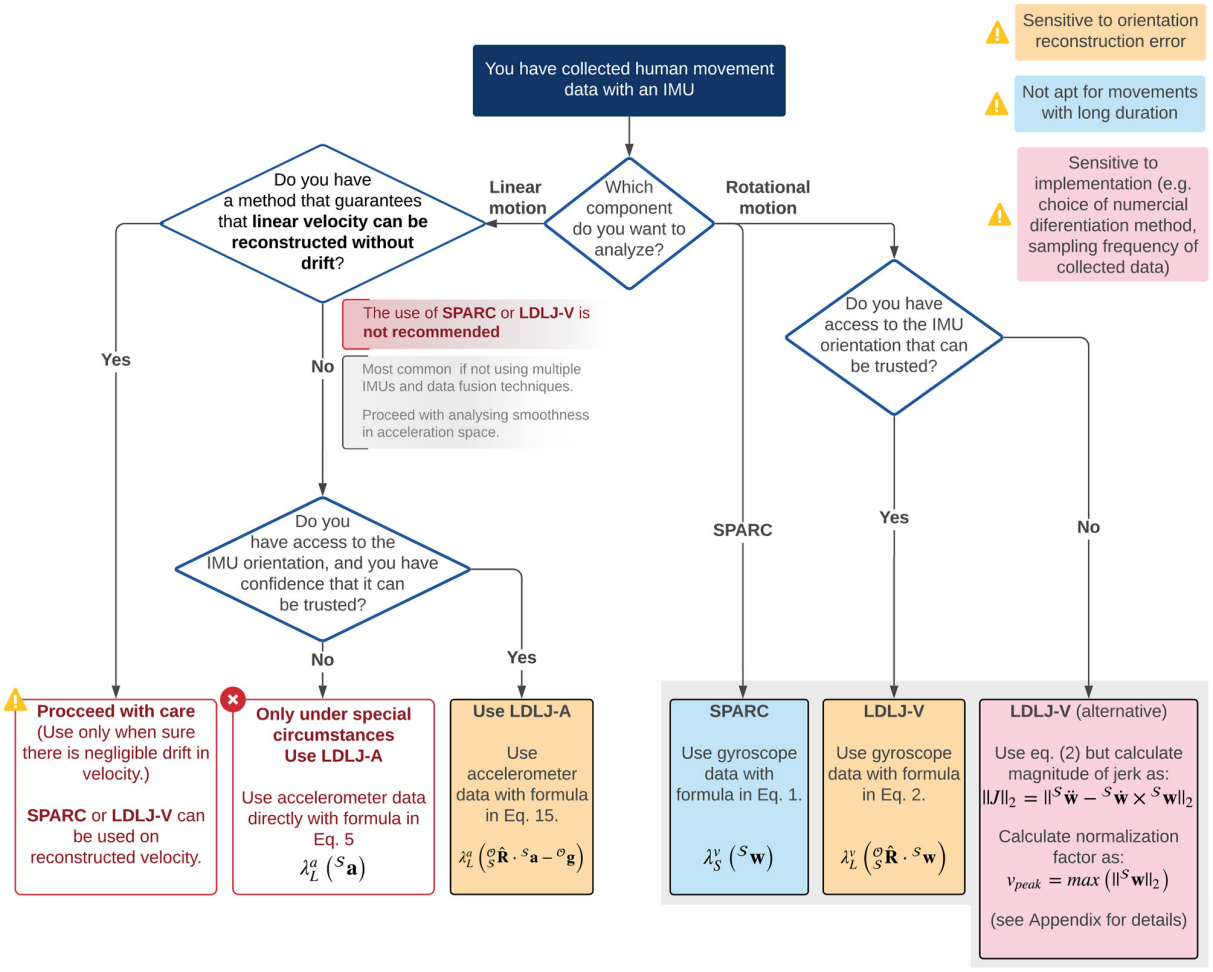
Summary diagram of recommendations for analyzing movement smoothness of discrete movements (i.e., with a clear start and end with zero velocity) using IMU data.

Quantification of movement smoothness is considered to be of great importance in movement sciences and biomechanics, to gain insights about the underlying mechanisms generating the observed motor behavior. IMUs are increasingly used to measure movement kinematics, but they do not directly measure position kinematics, have a time-varying frame of reference, and are noisy—which make deriving high-quality velocity and orientation kinematics in a frame of interest non-trivial. These issues put into question the appropriateness of IMU data to quantify movement smoothness in practice using SPARC and LDLJ-V. In this paper, we systematically analyzed the difficulties of using IMUs for movement smoothness analysis of translational and rotational movements. A summary of recommendations for the measures to use for analyzing movement smoothness using IMU data is in Figure 9 and discussed below.

### 5.1. Translational Movement Smoothness

Estimation of linear velocity is required to compute SPARC and LDLJ-V. In the case of an IMU, this requires integration of the accelerometer signals, which are affected by rotational movements of the sensor and are noisy. Even with good estimates of the IMU's orientation, reconstruction errors and accelerometer noise can translate into drift in the reconstructed linear velocity. This, in turn, affects the reliability of the smoothness measures (see Figures 7A,B). Thus, **SPARC and LDLJ-V should not be used on translational velocity kinematics obtained from an IMU**. There are, however, several techniques that can be used to obtain improved reconstructions of translational kinematics from fused information from multiple IMUs (Kok et al., 2017), which could enable the use of SPARC and LDLJ-V on IMU data. However, such pose reconstruction methods rely on application-specific models, constraints, and assumptions, which can introduce systematic biases in the reconstructed velocity. Thus, the same motion, processed by different algorithms, could result in significantly different movement smoothness estimates. To our knowledge, there have been no studies evaluating the sensitivity of smoothness measures on such translational velocity reconstruction algorithms from IMUs. Thus, at this point, estimating smoothness with SPARC and LDLJ-V using such algorithms are best avoided until their reliability is established.

Since jerk can be derived from acceleration data, jerk-based measures can potentially be calculated from acceleration with the appropriate modifications in the scaling factor. This resulted in the LDLJ-A proposed in Equation (16), which can be used with acceleration data. The analyses presented in this study show that the **LDLJ-A can be a good alternative to estimate translational movement smoothness from IMU acceleration data**. However, its use requires a reasonably well-reconstructed movement acceleration signal, and its accuracy is determined by the nature of the error in the IMU's orientation reconstruction, and the relative magnitude of the movement's translational acceleration with respect to gravity. This was illustrated with simulated (Figures 5C,D), and experimental data (Figure 8). Unfortunately, the extent of the orientation reconstruction error and the relative magnitude of linear acceleration with respect to gravity are often unknown in experimental or real-life data. In such a scenario, the SGR metric (Equation 21) can be used to provide some confidence in the smoothness estimates. Movements with an SGR of at least 1.05 were found to be less sensitive to different amounts of orientation reconstruction errors (Figures 5A, 8).

Estimating movement smoothness from an accelerometer without an estimate of its orientation should ideally be avoided except under the special circumstance where there is a good reason to believe that the sensor did not undergo much rotation, and the SGR is greater than 1.05 (the larger the SGR, the better the estimates). In this very specific case, one could potentially apply LDLJ-A directly on the accelerometer data ${\;}^{S}\text{a}\left(t\right)$.

### 5.2. Rotational Movement Smoothness

Analyzing rotational movements from IMU data is simpler than the translational case because gyroscopes directly measure rotational velocity and are unaffected by gravity. **SPARC can be applied to gyroscope data without any modifications**. LDLJ-V needs the gyroscope data to be corrected for sensor rotation (Equation 15), and thus, can be affected by reconstruction error. We note that there is another approach that can theoretically circumvent the need for orientation reconstruction (described in Appendix D), but this approach suffers from practical implementation issues which need further investigation.

In experimental data, we found that smoothness computed from IMU and MoCap data had better agreement when using LDLJ-V than SPARC. This is in contrast to our previous studies (Balasubramanian et al., 2012, 2015), in which we consistently found that SPARC is more reliable and less sensitive to noise than LDLJ-V. When looking closer at the SPARC results from section 4, we observed that the SPARC performance was poorer than LDLJ-V for less smooth (and also longer duration) movements. Movements that exhibited poorer levels of agreement had significant differences in spectral arc length, consequence of the integration of slight differences between the IMU and MoCap magnitude spectrum curves (at frequencies lower than 6 Hz). However, LDLJ-V was most probably insensitive to those differences because of the logarithmic transformation which compressed the differences in dimensionless jerk (the argument of the log function in Equation 16) between the IMU- and MoCap-based estimates. This problem was observed primarily for movements with a large number of submovements or undulations; such complex movements were not considered in our previous studies, and thus, this characteristic of SPARC was unnoticed. Future work could investigate possible transformations on SPARC (similar to the logarithm) that can alleviate this behavior.

One must not forget that the results for LDLJ-V and SPARC reported from experimental data only reflect the characteristics of the specific dataset presented here. It remains to be seen how these measures behave on a different set of movements. Since the correlation between the IMU- and MoCap-derived smoothness were very high for both SPARC and LDLJ-V, we cannot recommend the use of one measure over the other - both approaches seem like good candidates for analyzing movement smoothness of rotational movements measured by IMUs.

Furthermore, the good results from LDLJ-V in Figure 7E are in agreement with the simulated data. The results from the simulated data indicate that smoothness estimated from LDLJ-V has small errors for relatively unsmooth movements with small reconstruction errors (Figure 6A). The movements of the human subjects had lower smoothness (less than −10) relative to the simulated data (between −14 and −5). It is also possible that the IMU orientation reconstruction errors in the human data were small, which could also contribute to these results; the assumption of small orientation reconstruction error is also consistent with the good results seen with LDLJ-A for translational movements (Figure 7C); see also section 4.3). LDLJ-V can result in highly erroneous smoothness estimates for relatively smooth movements, and thus its use should be avoided for rotational movements if accurate orientation reconstruction cannot be guaranteed.

### 5.3. Limitations

It is crucial to mention some of the shortcomings of the current study to ensure the results are interpreted appropriately.

1. The work presented here is only an initial step toward standardizing methods for estimating **movement smoothness** from IMU measurements that is consistent with its definition, and with other movement measurement technologies. Further work is required before a good, well-accepted method can be established in the field.
2. The formulations presented here are only valid for discrete movements, i.e., movements that start and end with a static posture (Hogan and Sternad, 2007). Further work is required to establish methods for computing movement smoothness of rhythmic movements, such as walking.
3. The preliminary validation of the different proposed approaches in this work are based on a limited set of simulated and experimental data. Thus, it is possible that part of the study outcomes are specific to these datasets. We attempted to mitigate this by including a broad range of behaviors in the simulated and experimental movements, resulting in a wide range of smoothness values. The movement simulated in this study have characteristics (number of submovements, duration etc.) that are commonly observed in data from patients with movement impairments (Rohrer et al., 2004). The smoothness values of most of the simulated movements shared the same range of values are the patient data presented in the paper (-6 to -4 for the LDLJ-A). Nevertheless, the recommendations made in this study must be validated using a larger experimental dataset with more subjects and a wider range of movement tasks.
4. The tracking of rotational movements with MoCap was found to be quite noisy, which could have been the reason for the bias and the wider limits of agreement for SPARC and LDLJ-V. A future validation study could quantify the noise characteristics of IMU and MoCap systems to better evaluate their effects on smoothness measures.

## 6. Conclusion

In this paper, we carried out a systematic analysis to identify appropriate methods to analyze smoothness of discrete movements measured by an IMU, that are consistent with analyses performed with measurement technology in which movement velocity relative to a fixed reference frame can be reliably obtained. Our results suggest that there is no single optimal approach for analyzing both translational and rotational movements measured by an IMU. The most appropriate method for estimating movement smoothness depends on two factors: (a) the type of movement (rotational or translational), and (b) the accuracy of the estimate of the IMU's orientation. Based on our analysis, the recommendations for analyzing translational and rotational motions for smoothness analysis are summarized in Figure 9. Future studies must evaluate these recommendations on larger datasets consisting of different types of movements.

## Data Availability Statement

The code used for the generation and analysis of the simulated data in this paper is available at: https://github.com/siva82kb/smoothness_from_imu. Experimental data belong to another study (as specified in section Discussion), and cannot be made available with this article. Data might be available under reasonable request by contacting the study investigators.

## Author Contributions

All authors contributed to the conception of the ideas and the theoretical aspects of the work. CS and AM-C worked on the experimental setup and data collection used for validating the various smoothness measures. AM-C analyzed the experimental data. SB worked on the simulation analysis of the different smoothness measures. All authors contributed equally to the interpretation of results, writing and reviewing of the manuscript.

## Conflict of Interest

The authors declare that the research was conducted in the absence of any commercial or financial relationships that could be construed as a potential conflict of interest.

## Notation

*r* : scalar (lowercase italic letter)
**r** : vector (lowercase bold letter)
**R** : matrix (uppercase bold letter)
A : coordinate frame (calligraphy uppercase letter)
*A* : origin of coordinate frame A
${\;}^{C}{\text{x}}_{AB}$ : position vector of point *B* w.r.t point *A* expressed in frame C
${\;}^{C}\omega_{AB}$ : angular velocity vector of frame B w.r.t frame A expressed in frame C
${\;}_{B}^{A}\text{R }$ : rotation matrix that maps vectors expressed in frame B to frame A
*t* : time
*t*_1_ : start time of a discrete movement (onset)
*t*_2_ : stop time of a discrete movement (termination)
*g* : gravitational acceleration constant (9.81*m*/*s*^2^)
$\lambda_{*}^{†}$ : smoothness measure using method * ∈ {*S, L*}, where *S* and *L* correspond to SPARC and LDLJ respectively, and evaluated from data type † ∈ {*p, v, a, j*}, where *p*, *v*, *a*, and *j* correspond to position, velocity, acceleration and jerk, respectively.
