# Supplementary material for: Estimating Movement Smoothness From Inertial Measurement Units
Source: Front Bioeng Biotechnol. 2021 Jan 14;8:558771. doi: 10.3389/fbioe.2020.558771 (PMC7841375; doi:10.3389/fbioe.2020.558771)
Supplement: Supplementary file 1 [file Presentation_1.pdf]

Estimating Movement Smoothness from Inertial Measurement Units: Supplementary Material

Alejandro Melendez-Calderon Camila Shirota
Sivakumar Balasubramanian

In this supplementary document, all equations and figures are referenced with Roman numerals. Any reference using the Arabic numerals refers to contect in the main document.

Appendix A

Kinematics of a segment moving relative to another

Translational motion - The linear velocity of $\mathcal{S}$ relative to $\mathcal{B}$ (Fig. 2 in the main text) is given by:

$$\begin{aligned} {}^{\mathcal{O}}\mathbf{x}_{OS} &= {}^{\mathcal{O}}\mathbf{x}_{OB} + {}^{\mathcal{O}}\mathbf{x}_{BS} \\ &= {}^{\mathcal{O}}\mathbf{x}_{OB} + {}^{\mathcal{O}}_{\mathcal{B}}\mathbf{R}\,{}^{\mathcal{B}}\mathbf{x}_{BS} \qquad \text{(I)} \\ \implies {}^{\mathcal{O}}\dot{\mathbf{x}}_{OS} &= {}^{\mathcal{O}}\dot{\mathbf{x}}_{OB} + {}^{\mathcal{O}}_{\mathcal{B}}\dot{\mathbf{R}}\,{}^{\mathcal{B}}\mathbf{x}_{BS} + {}^{\mathcal{O}}_{\mathcal{B}}\mathbf{R}\,{}^{\mathcal{B}}\dot{\mathbf{x}}_{BS} \qquad \text{(II)} \\ \therefore \quad {}^{\mathcal{B}}\dot{\mathbf{x}}_{BS} &= {}^{\mathcal{B}}_{\mathcal{O}}\mathbf{R}\,\left({}^{\mathcal{O}}\dot{\mathbf{x}}_{OS} - {}^{\mathcal{O}}\dot{\mathbf{x}}_{OB} - {}^{\mathcal{O}}_{\mathcal{B}}\dot{\mathbf{R}}\,{}^{\mathcal{B}}\mathbf{x}_{BS}\right). \qquad \text{(III)} \end{aligned}$$

Thus, ${}^{\mathcal{B}}\dot{\mathbf{x}}_{BS}$ is related to ${}^{\mathcal{O}}\dot{\mathbf{x}}_{OS}$ through the translational ${}^{\mathcal{O}}_{\mathcal{B}}\mathbf{R}\,{}^{\mathcal{B}}\dot{\mathbf{x}}_{BS}$ and rotational ${}^{\mathcal{O}}_{\mathcal{B}}\dot{\mathbf{R}}\,{}^{\mathcal{B}}\mathbf{x}_{BS}$ movement components of $\mathcal{B}$ relative to $\mathcal{O}$.

The linear acceleration of $\mathcal{S}$ with respect to $\mathcal{O}$ is obtained by differentiating Eq. (II):

$${}^{\mathcal{O}}\ddot{\mathbf{x}}_{OS} = {}^{\mathcal{O}}_{\mathcal{B}}\mathbf{R}\,{}^{\mathcal{B}}\ddot{\mathbf{x}}_{BS} + {}^{\mathcal{O}}\ddot{\mathbf{x}}_{OB} + 2\,{}^{\mathcal{O}}_{\mathcal{B}}\dot{\mathbf{R}}\,{}^{\mathcal{B}}\dot{\mathbf{x}}_{BS} + {}^{\mathcal{O}}_{\mathcal{B}}\ddot{\mathbf{R}}\,{}^{\mathcal{B}}\mathbf{x}_{BS} \qquad \text{(IV)}$$

Combining Eqs. 3 and IV to get the accelerometer signal ${}^{\mathcal{S}}\mathbf{a}$:

$$\begin{aligned} {}^{\mathcal{S}}\mathbf{a} &= {}^{\mathcal{S}}_{\mathcal{O}}\mathbf{R}\,\left({}^{\mathcal{O}}_{\mathcal{B}}\mathbf{R}\,{}^{\mathcal{B}}\ddot{\mathbf{x}}_{BS} + {}^{\mathcal{O}}\ddot{\mathbf{x}}_{OB} + 2\,{}^{\mathcal{O}}_{\mathcal{B}}\dot{\mathbf{R}}\,{}^{\mathcal{B}}\dot{\mathbf{x}}_{BS} + {}^{\mathcal{O}}_{\mathcal{B}}\ddot{\mathbf{R}}\,{}^{\mathcal{B}}\mathbf{x}_{BS} + {}^{\mathcal{O}}\mathbf{g}\right) \\ &= {}^{\mathcal{S}}_{\mathcal{B}}\mathbf{R}\,{}^{\mathcal{B}}\ddot{\mathbf{x}}_{BS} + {}^{\mathcal{S}}\mathbf{g} + {}^{\mathcal{S}}\ddot{\mathbf{x}}_{OB} + 2\,{}^{\mathcal{S}}_{\mathcal{O}}\mathbf{R}\,{}^{\mathcal{O}}_{\mathcal{B}}\dot{\mathbf{R}}\,{}^{\mathcal{B}}\dot{\mathbf{x}}_{BS} + {}^{\mathcal{S}}_{\mathcal{O}}\mathbf{R}\,{}^{\mathcal{O}}_{\mathcal{B}}\ddot{\mathbf{R}}\,{}^{\mathcal{B}}\mathbf{x}_{BS} \qquad \text{(V)} \end{aligned}$$

Rotational motion - The angular velocity of $\mathcal{S}$ with respect to $\mathcal{B}$ is given by:

$${}^{\mathcal{B}}\omega_{\mathcal{BS}} = {}^{\mathcal{B}}_{\mathcal{O}}\mathbf{R}\,\left({}^{\mathcal{O}}\omega_{\mathcal{OS}} - {}^{\mathcal{O}}\omega_{\mathcal{OB}}\right). \qquad \text{(VI)}$$

Combining Eqs. VI and 4, we can obtain ${}^{\mathcal{B}}\omega_{\mathcal{BS}}$ from the IMU's gyroscope signal ${}^{\mathcal{S}}\mathbf{w}$

$$\begin{aligned} {}^{\mathcal{B}}\omega_{\mathcal{BS}} &= {}^{\mathcal{B}}_{\mathcal{O}}\mathbf{R}\,{}^{\mathcal{O}}_{\mathcal{S}}\mathbf{R}\,{}^{\mathcal{S}}\mathbf{w} - {}^{\mathcal{B}}\omega_{\mathcal{OB}} \qquad \text{(VII)} \\ \therefore \quad {}^{\mathcal{S}}\mathbf{w} &= {}^{\mathcal{S}}_{\mathcal{O}}\mathbf{R}\,{}^{\mathcal{O}}_{\mathcal{B}}\mathbf{R}\,\left({}^{\mathcal{B}}\omega_{\mathcal{BS}} + {}^{\mathcal{B}}\omega_{\mathcal{OB}}\right) \qquad \text{(VIII)} \end{aligned}$$

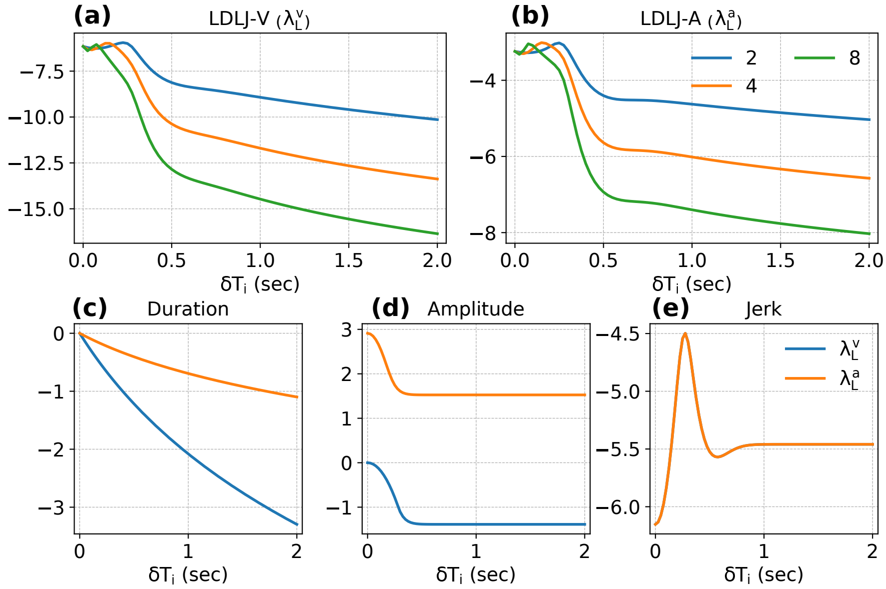

Figure I: Consistency of λ_L^a compared to λ_L^v. Smoothness of movements with 2 (blue), 4 (orange) or 8 (green) via points, estimated with (a) λ_L^v and (b) λ_L^a, as a function of inter-submovement interval. The response of the three individual terms (c) Duration (T), (d) Amplitude (A), and (e) Jerk (J) as a function of (δT_i) for λ_L^v and λ_L^a.

Appendix B

Analysis of the properties of LDLJ-A

In this paper, we defined a modified version of LDLJ-V derived from movement acceleration (LDLJ-A or λ_L^a, Eq. 16). λ_L^a and λ_L^v (Eq. 2) differ only in their scaling factor $\frac{(t_2-t_1)}{a_{peak}^2}$ and $\frac{(t_2-t_1)^3}{v_{peak}^2}$, respectively, since

$$\int_{t_1}^{t_2} \left\| \frac{d\mathbf{a}}{dt} \right\|_2^2 dt = \int_{t_1}^{t_2} \left\| \frac{d^2\mathbf{v}}{dt^2} \right\|_2^2 dt.$$

Thus,

$$\lambda_L^v(\mathbf{v}) = \lambda_L^a(\mathbf{a}) - 2\ln(t_2 - t_1) - 2\ln\left(\frac{a_{peak}}{v_{peak}}\right).$$

This relationship is non-linear due to the $2\ln\left(\frac{a_{peak}}{v_{peak}}\right)$ factor.

Consistency of LDLJ-A. The consistency of the LDLJ-V and LDLJ-A measures were evaluated using simulated 1D movements, which were generated as a sum of a varying number of submovements with varying inter-submovement intervals. The movement velocity and acceleration profiles of these 1D movements were of the form,

$$v(t; N_s, \delta T_i) = \sum_{n=0}^{N_s-1} \psi(t - n\delta T_i)$$
$$a(t; N_s, \delta T_i) = \frac{d}{dt} v(t; N_s, \delta T_i)$$

where $v(\cdot)$ and $a(\cdot)$ are the velocity and acceleration of the movement, respectively, $\psi(t) = e^{-25(t-0.5)^2}$ is a smooth submovement, $\delta T_i \in \left\{0.001i \;\middle|\; i \in \mathbb{N} \,\text{and}\, 0 \leq i \leq 2000\right\}$ are the inter-submovement intervals, and $N_s \in \{2, 4, 8\}$ is the number of submovements. The smoothness values of this set of movements was evaluated using the LDLJ-V and LDLJ-A applied to the velocity and acceleration data respectively.

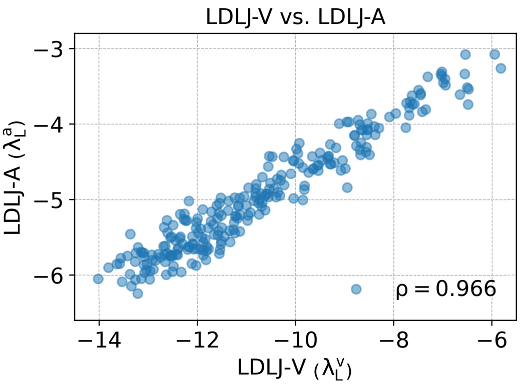

Figure II: Scatter plot of λ_L^v and λ_L^a for a set of movements with varying number of submovements. The scatter plot shows that there is a fair correlation between the smoothness values reported by the two movements on the same movement data, but there is no one-to-one relationship.

λ_L^v and λ_L^a have roughly similar responses to changes in δT_i, and show approximately monotonic responses to the change in submovement characteristics (Fig. I). However, in general, for the same movement, smoothness values λ_L^v and λ_L^a will not be the same.

LDLJ-A and LDLJ-V cannot be interchanged. Although λ_L^v and λ_L^a do not provide the same smoothness values, they could still be used interchangeably if they would report the same relative smoothness between any two movements. Mathematically, if there is be an isomorphism between the measures, that is, given movements M_1 and M_2 with velocity profiles $\mathbf{v}_1$ and $\mathbf{v}_2$, respectively, then

$$\lambda_L^v(\mathbf{v}_1) > \lambda_L^v(\mathbf{v}_2) \iff \lambda_L^a(\dot{\mathbf{v}}_1) > \lambda_L^a(\dot{\mathbf{v}}_2), \forall \mathbf{v}_1, \mathbf{v}_2$$

To check if such an isomorphism exists between λ_L^v and λ_L^a, we simulated a set of discrete, minimum jerk, 3D movements through via-points. The generation of these movements was posed as a constrained optimization problem and was solved using the CVXPY solver in Python, similar to the approach used in [2]:

$$\begin{aligned} \underset{\mathbf{x}}{\text{minimize}} \quad & \|\mathbf{D}\mathbf{x}\|_2^2 \\ \text{subject to } & \mathbf{A}\mathbf{x} = \mathbf{b} \end{aligned}$$

where $\mathbf{x}$ is a stacked vector of positions corresponding to the movement trajectory, $\mathbf{D}$ is the matrix performing the triple derivative to obtain movement jerk from $\mathbf{x}$, and $\mathbf{A}$ and $\mathbf{b}$ represent the spatio-temporal constraints corresponding to initial, final, and via-points position, and the zero initial, and final velocity and acceleration constraints. The solution $\mathbf{x}_m$ obtained using this procedure would possess the minimum squared jerk $\|\mathbf{D}\mathbf{x}\|_2^2$ for the given spatial and temporal constraints.

We simulated movements with initial position $\mathbf{x}_i = \begin{bmatrix} 0 & 0 & 0 \end{bmatrix}^\top$, final position $\mathbf{x}_f = \begin{bmatrix} 0 & 1 & 0 \end{bmatrix}^\top$, and duration $T = 1s$. A set of N via-point spatio-temporal constraints $\{\mathbf{x}_n^v, t_n\}_{n=1}^N$ were randomly generated, where the nth via-point $\mathbf{x}_n^v$ is traversed at time t_n. A set X_m of 251 minimum jerk trajectories were generated, corresponding to: (a) 25 different spatio-temporal constraints for each of 10 different values of N; and (b) one movement without any via-points. The sampling period was $\delta t_s = 0.001s$, resulting in 1000 sample points per movement.

The smoothness of the movements in X_m estimated using λ_L^v and λ_L^a were fairly well correlated (Fig. II), but not isomorphic. This implies that the two measures have slightly different structures, and one cannot be exactly obtained from the other.

Appendix C

SPARC on translational velocity and acceleration data

The SPARC measure was also applied to the simulated data (from Appendix B) to evaluate the measure's consistency when estimated from movement velocity or acceleration (Fig. III). Similar to [1], there was a reasonably consistent response when using velocity data (Fig. III(a)). On the other hand, when movement acceleration is used (Fig. III(b)), it does not result in a consistent response (i.e., a monotonic response to change in inter-submovement interval).

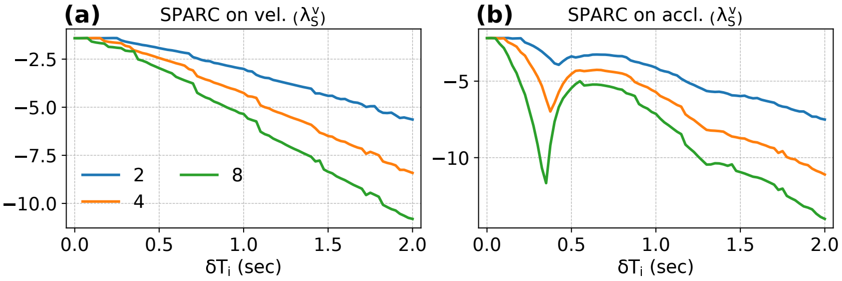

Figure III: Consistency of λ_S^v applied to (a) movement velocity and (b) movement acceleration.

This difference in behavior is due to the definition of SPARC: it was inspired by the idea of movement planning and execution by combining submovements. SPARC measures the level of dispersion of a signal in time (e.g., relative periods of movement and arrest). This measure of temporal dispersion acts as a reasonable measure of smoothness only when it is applied on the velocity, because velocity dispersion is strongly related to movement intermittency. Temporal dispersion in all other kinematic variables do not provide a unique measure of intermittency.

Appendix D

Calculating magnitude of linear jerk from IMU signals without orientation estimation

The derivative of the accelerometer signal ${}^{S}\mathbf{a}$ is given by (from Eqs. 3 and 4):

$$\begin{aligned}
{}^{S}\dot{\mathbf{a}} &= \frac{d}{dt}\left({}^{S}_{O}\mathbf{R}\ \left({}^{O}\ddot{\mathbf{x}}_{OS} + {}^{O}\mathbf{g}\right)\right) \\
&= {}^{S}_{O}\dot{\mathbf{R}}\ \left({}^{O}\ddot{\mathbf{x}}_{OS} + {}^{O}\mathbf{g}\right) + {}^{S}_{O}\mathbf{R}\ {}^{O}\dddot{\mathbf{x}}_{OS} \\
\therefore \quad {}^{S}_{O}\mathbf{R}\ {}^{O}\dddot{\mathbf{x}}_{OS} &= {}^{S}\dot{\mathbf{a}} - {}^{S}_{O}\dot{\mathbf{R}}\ \left({}^{O}\ddot{\mathbf{x}}_{OS} + {}^{O}\mathbf{g}\right) \\
&= {}^{S}\dot{\mathbf{a}} - {}^{S}_{O}\dot{\mathbf{R}}\ {}^{O}_{S}\mathbf{R}\ {}^{S}\mathbf{a} \\
&= {}^{S}\dot{\mathbf{a}} - \left[{}^{S}\omega_{SO}\right]_{\times}\ {}^{S}_{O}\mathbf{R}\ {}^{O}_{S}\mathbf{R}\ {}^{S}\mathbf{a} \\
&= {}^{S}\dot{\mathbf{a}} - {}^{S}\omega_{SO} \times {}^{S}\mathbf{a} \\
&= {}^{S}\dot{\mathbf{a}} - {}^{S}\mathbf{a} \times {}^{S}\omega_{OS} \\
&= {}^{S}\dot{\mathbf{a}} - {}^{S}\mathbf{a} \times {}^{S}\mathbf{w}
\end{aligned} \tag{IX}$$

where the matrix $\left[{}^{S}\omega_{SO}\right]_{\times}$ is skew-symmetric.

Because the operator $\|\ \|_2$ is rotation invariant, the magnitude of the linear jerk is given by:

$$\|{}^{O}\dddot{\mathbf{x}}_{OS}\|_2 = \|{}^{S}_{O}\mathbf{R}\ {}^{O}\dddot{\mathbf{x}}_{OS}\|_2 = \|{}^{S}\dot{\mathbf{a}} - {}^{S}\mathbf{a} \times {}^{S}\mathbf{w}\|_2 \tag{X}$$

It is important to note that, in practice, the term ${}^{S}\dot{\mathbf{a}}$ is computed from numerical differentiation. Subtracting the correction term ${}^{S}\mathbf{a} \times {}^{S}\mathbf{w}$, which is derived from the raw IMU signals, is

affected by misalignment in time and different sampling of peaks. In simulated data, we found that, in some cases, this can lead to erroneous estimation of the translational jerk. Further investigation is needed before general recommendations can be provided for this approach.

Calculating magnitude of angular jerk from IMU signals without rotation estimation

Similar to translational jerk, the absolute angular jerk can be computed directly from IMU data. The second derivative of the gyroscope signal ${}^{s}\mathbf{w}$ is given by:

$$\begin{aligned}
{}^{s}\ddot{\mathbf{w}} &= \frac{d^2}{dt^2}\left({}^{s}_{o}\mathbf{R}\,{}^{o}\omega_{os}\right) \\
&= \frac{d}{dt}\left({}^{s}_{o}\dot{\mathbf{R}}\,{}^{o}\omega_{os} + {}^{s}_{o}\mathbf{R}\,{}^{o}\dot{\omega}_{os}\right) \\
&= {}^{s}_{o}\ddot{\mathbf{R}}\,{}^{o}\omega_{os} + 2\,{}^{s}_{o}\dot{\mathbf{R}}\,{}^{o}\dot{\omega}_{os} + {}^{s}_{o}\mathbf{R}\,{}^{o}\ddot{\omega}_{os} \\
\therefore \quad {}^{s}_{o}\mathbf{R}\,{}^{o}\ddot{\omega}_{os} &= {}^{s}\ddot{\mathbf{w}} - {}^{s}_{o}\ddot{\mathbf{R}}\,{}^{o}\omega_{os} - 2\,{}^{s}_{o}\dot{\mathbf{R}}\,{}^{o}\dot{\omega}_{os} \\
&= {}^{s}\ddot{\mathbf{w}} - {}^{s}\dot{\omega}_{so} \times {}^{s}\omega_{os} - 2\left({}^{s}\omega_{so} \times {}^{s}\dot{\omega}_{os}\right) \\
&= {}^{s}\ddot{\mathbf{w}} - {}^{s}\dot{\omega}_{os} \times {}^{s}\omega_{os} \\
&= {}^{s}\ddot{\mathbf{w}} - {}^{s}\dot{\mathbf{w}} \times {}^{s}\mathbf{w}. \qquad \text{(XI)}
\end{aligned}$$

Thus, the magnitude of the absolute angular jerk can be calculated as:

$$\left\|{}^{o}\ddot{\omega}_{os}\right\|_2 = \left\|{}^{s}_{o}\mathbf{R}\,{}^{o}\ddot{\omega}_{os}\right\|_2 = \left\|{}^{s}\ddot{\mathbf{w}} - {}^{s}\dot{\mathbf{w}} \times {}^{s}\mathbf{w}\right\|_2. \qquad \text{(XII)}$$

In practice, numerical differentiation would be necessary to obtain ${}^{s}\ddot{\mathbf{w}}$ and ${}^{s}\dot{\mathbf{w}}$, which could result in to erroneous estimates. Indeed, the formulation in Eq. XII was found to be sensitive to practical implementation issues (e.g. choice of numerical differentiation methods, sampling frequency, etc.). Therefore, further investigation is needed before general recommendations can be made for this formulation to be used in movement smoothness analyses.

Appendix E

Errors in LDLJ-A smoothness measured from reconstructed movements

Errors in orientation reconstruction $\delta\mathbf{R}$ result in errors in estimated movement acceleration ${}^{o}\hat{\ddot{\mathbf{x}}}_{os} = \delta\mathbf{R}\,{}^{o}\ddot{\mathbf{x}}_{os} + (\delta\mathbf{R} - \mathbf{I})\,{}^{o}\mathbf{g}$. These errors are reflected in the jerk integral $\hat{I}_{\mathrm{j}}$ and the peak acceleration $\hat{a}_{peak}$, thus affecting LDLJ-A. In summary:

- When $\delta\mathbf{R}$ is constant over time, then there is zero error in the smoothness estimate (Eqs. XIV and XVII).
- When $\delta\mathbf{R} \neq \mathbf{0}$, movements with larger acceleration/jerk are likely to have smaller errors in their smoothness estimates (Eqs. XV-XVI and Eqs. XIX-XX).
- Small values of $\delta\dot{\mathbf{R}}$ result in small errors in the jerk integral term.
- When $\Delta_{\delta\mathbf{R}}$ (Eq. 20) is small, errors in peak acceleration are likely to be small.

In the rest of this section, we provide the mathematical details supporting these statements. **Error in the jerk integral term**. Consider a movement M with true acceleration ${}^{\mathcal{O}}\ddot{\mathbf{x}}_{OS}$ and reconstructed acceleration ${}^{\mathcal{O}}\hat{\ddot{\mathbf{x}}}_{OS}$. From Eq. 14, the derivative of the reconstructed acceleration is given by ${}^{\mathcal{O}}\hat{\dddot{\mathbf{x}}}_{OS} = \delta\mathbf{R}\,{}^{\mathcal{O}}\dddot{\mathbf{x}}_{OS} + \delta\dot{\mathbf{R}}\,\left({}^{\mathcal{O}}\ddot{\mathbf{x}}_{OS} + {}^{\mathcal{O}}\mathbf{g}\right)$, thus

$$\begin{aligned} \left\|{}^{\mathcal{O}}\hat{\dddot{\mathbf{x}}}_{OS}\right\|_2^2 &= \left\|{}^{\mathcal{O}}\dddot{\mathbf{x}}_{OS}\right\|_2^2 + \left\|\delta\dot{\mathbf{R}}\,\left({}^{\mathcal{O}}\ddot{\mathbf{x}}_{OS} + {}^{\mathcal{O}}\mathbf{g}\right)\right\|_2^2 + 2\,{}^{\mathcal{O}}\dddot{\mathbf{x}}_{OS}^{\top}\,\delta\mathbf{R}^{\top}\,\delta\dot{\mathbf{R}}\left({}^{\mathcal{O}}\ddot{\mathbf{x}}_{OS} + {}^{\mathcal{O}}\mathbf{g}\right) \\ \implies \left\|{}^{\mathcal{O}}\hat{\dddot{\mathbf{x}}}_{OS}\right\|_2^2 - \left\|{}^{\mathcal{O}}\dddot{\mathbf{x}}_{OS}\right\|_2^2 &= \left\|\delta\dot{\mathbf{R}}\,\left({}^{\mathcal{O}}\ddot{\mathbf{x}}_{OS} + {}^{\mathcal{O}}\mathbf{g}\right)\right\|_2^2 + 2\,{}^{\mathcal{O}}\dddot{\mathbf{x}}_{OS}^{\top}\,\delta\mathbf{R}^{\top}\,\delta\dot{\mathbf{R}}\left({}^{\mathcal{O}}\ddot{\mathbf{x}}_{OS} + {}^{\mathcal{O}}\mathbf{g}\right) \end{aligned} \tag{XIII}$$

Thus, the difference between the true and the reconstructed squared jerk magnitudes consists of:

- $\left\|\delta\dot{\mathbf{R}}\,\left({}^{\mathcal{O}}\ddot{\mathbf{x}}_{OS} + {}^{\mathcal{O}}\mathbf{g}\right)\right\|_2^2$: the rate of change of reconstructed acceleration due to time varying rotational error $\delta\dot{\mathbf{R}}$.
- $2\,{}^{\mathcal{O}}\dddot{\mathbf{x}}_{OS}^{\top}\,\delta\mathbf{R}^{\top}\,\delta\dot{\mathbf{R}}\left({}^{\mathcal{O}}\ddot{\mathbf{x}}_{OS} + {}^{\mathcal{O}}\mathbf{g}\right)$: Inner product between the rotational error $\delta\mathbf{R}\,{}^{\mathcal{O}}\dddot{\mathbf{x}}_{OS}$ and the time-varying rotational error $\delta\dot{\mathbf{R}}\left({}^{\mathcal{O}}\ddot{\mathbf{x}}_{OS} + {}^{\mathcal{O}}\mathbf{g}\right)$.

Let δJ be the relative error between the mean squared magnitudes of the true and reconstructed jerk magnitudes. Using Eq. XIII:

$$\begin{aligned} \delta J &= \frac{\left\|{}^{\mathcal{O}}\hat{\dddot{\mathbf{x}}}_{OS}\right\|_2^2 - \left\|{}^{\mathcal{O}}\dddot{\mathbf{x}}_{OS}\right\|_2^2}{\left\|{}^{\mathcal{O}}\dddot{\mathbf{x}}_{OS}\right\|_2^2} = \frac{\left\|\delta\dot{\mathbf{R}}\,\left({}^{\mathcal{O}}\ddot{\mathbf{x}}_{OS} + {}^{\mathcal{O}}\mathbf{g}\right)\right\|_2^2}{\left\|{}^{\mathcal{O}}\dddot{\mathbf{x}}_{OS}\right\|_2^2} + \frac{2\,{}^{\mathcal{O}}\dddot{\mathbf{x}}_{OS}^{\top}\,\delta\mathbf{R}^{\top}\,\delta\dot{\mathbf{R}}\left({}^{\mathcal{O}}\ddot{\mathbf{x}}_{OS} + {}^{\mathcal{O}}\mathbf{g}\right)}{\left\|{}^{\mathcal{O}}\dddot{\mathbf{x}}_{OS}\right\|_2^2} \\ &= \frac{\left\|\delta\dot{\mathbf{R}}\,\left({}^{\mathcal{O}}\ddot{\mathbf{x}}_{OS} + {}^{\mathcal{O}}\mathbf{g}\right)\right\|_2^2}{\left\|{}^{\mathcal{O}}\dddot{\mathbf{x}}_{OS}\right\|_2^2} + 2\left(\delta\mathbf{R}\,\frac{{}^{\mathcal{O}}\dddot{\mathbf{x}}_{OS}}{\left\|{}^{\mathcal{O}}\dddot{\mathbf{x}}_{OS}\right\|_2}\right)^{\top}\,\frac{\delta\dot{\mathbf{R}}\,\left({}^{\mathcal{O}}\ddot{\mathbf{x}}_{OS} + {}^{\mathcal{O}}\mathbf{g}\right)}{\left\|{}^{\mathcal{O}}\dddot{\mathbf{x}}_{OS}\right\|_2}. \end{aligned} \tag{XIV}$$

Thus, when the reconstruction error is fixed and does not vary over time, i.e. $\delta\dot{\mathbf{R}} = \mathbf{0}$, then $\delta J = 0$. When **the orientation reconstruction error tends towards zero**, then $\delta\dot{\mathbf{R}} \to \mathbf{0}$ (assuming no discontinuities in $\delta\mathbf{R}$), and it can be shown that δJ tends to 0, i.e. $\lim_{\delta\mathbf{R}\to\mathbf{I}} \delta J = 0$.

However, when there is time-varying reconstruction error $\delta\mathbf{R} \neq \mathbf{I}$ and $\delta\dot{\mathbf{R}} \neq \mathbf{0}$, then δJ will be non-zero and its magnitude will be determined by acceleration and jerk magnitudes of the movement:

- **When ${}^{\mathcal{O}}\ddot{\mathbf{x}}_{OS}$ and ${}^{\mathcal{O}}\dddot{\mathbf{x}}_{OS}$ are small**, δJ is dominated by gravity:

$$\delta J \approx \frac{\left\|\delta\dot{\mathbf{R}}\,{}^{\mathcal{O}}\mathbf{g}\right\|_2^2}{\left\|{}^{\mathcal{O}}\dddot{\mathbf{x}}_{OS}\right\|_2^2} + 2\left(\delta\mathbf{R}\,\frac{{}^{\mathcal{O}}\dddot{\mathbf{x}}_{OS}}{\left\|{}^{\mathcal{O}}\dddot{\mathbf{x}}_{OS}\right\|_2}\right)^{\top}\,\frac{\delta\dot{\mathbf{R}}\,{}^{\mathcal{O}}\mathbf{g}}{\left\|{}^{\mathcal{O}}\dddot{\mathbf{x}}_{OS}\right\|_2}. \tag{XV}$$

  δJ can be quite large depending on $\left\|{}^{\mathcal{O}}\dddot{\mathbf{x}}_{OS}\right\|_2^2$. Thus, incorrectly reconstructed, slow movements can have large errors in δJ and thus in the LDLJ-A smoothness estimates.

- **Movements with larger acceleration/jerk reduce the effect of gravity**. Let δJ in Eq. XIV be the error for a movement M measured using an IMU. If the overall amplitude of movement M was larger (no change in duration), such that the acceleration and jerk of this larger movement are $A\,{}^{\mathcal{O}}\ddot{\mathbf{x}}_{OS}$ and $A\,{}^{\mathcal{O}}\dddot{\mathbf{x}}_{OS}$, respectively, where $A > 1$ is the scale of the movement. The relative error in the jerk magnitude δJ_A will then be,

$$\begin{aligned} \delta J_A &= \frac{\left\|\delta\dot{\mathbf{R}}\,\left(A\,{}^{\mathcal{O}}\ddot{\mathbf{x}}_{OS} + {}^{\mathcal{O}}\mathbf{g}\right)\right\|_2^2}{\left\|A\,{}^{\mathcal{O}}\dddot{\mathbf{x}}_{OS}\right\|_2^2} + 2\left(\delta\mathbf{R}\,\frac{A\,{}^{\mathcal{O}}\dddot{\mathbf{x}}_{OS}}{A\,\left\|{}^{\mathcal{O}}\dddot{\mathbf{x}}_{OS}\right\|_2}\right)^{\top}\,\frac{\delta\dot{\mathbf{R}}\,\left(A\,{}^{\mathcal{O}}\ddot{\mathbf{x}}_{OS} + {}^{\mathcal{O}}\mathbf{g}\right)}{\left\|A\,{}^{\mathcal{O}}\dddot{\mathbf{x}}_{OS}\right\|_2} \\ &= \frac{\left\|\delta\dot{\mathbf{R}}\,\left({}^{\mathcal{O}}\ddot{\mathbf{x}}_{OS} + {}^{\mathcal{O}}\mathbf{g}/A\right)\right\|_2^2}{\left\|{}^{\mathcal{O}}\dddot{\mathbf{x}}_{OS}\right\|_2^2} + 2\left(\delta\mathbf{R}\,\frac{{}^{\mathcal{O}}\dddot{\mathbf{x}}_{OS}}{\left\|{}^{\mathcal{O}}\dddot{\mathbf{x}}_{OS}\right\|_2}\right)^{\top}\,\frac{\delta\dot{\mathbf{R}}\,\left({}^{\mathcal{O}}\ddot{\mathbf{x}}_{OS} + {}^{\mathcal{O}}\mathbf{g}/A\right)}{\left\|{}^{\mathcal{O}}\dddot{\mathbf{x}}_{OS}\right\|_2} \end{aligned} \tag{XVI}$$

- **Shorter movements will have lower error in the jerk integral term**. For two movements with the same average relative error in the jerk magnitude, the shorter movement will have an overall lower error because of shorter period of integration.

Error in the peak acceleration term. The value of a_{peak} is computed from the magnitude of the mean subtracted reconstructed acceleration (Eq. 16). The ratio between the magnitude of the reconstructed and the true acceleration is:

$$\begin{aligned}\delta a &= \frac{\left\| {}^{O}\hat{\ddot{\mathbf{x}}}_{OS} \right\|_2}{\left\| {}^{O}\ddot{\mathbf{x}}_{OS} \right\|_2} \\ &= \frac{1}{\left\| {}^{O}\ddot{\mathbf{x}}_{OS} \right\|_2} \left\| \delta\mathbf{R}\ {}^{O}\ddot{\mathbf{x}}_{OS} - \frac{1}{\Delta t}\int_{t_1}^{t_2} \delta\mathbf{R}\ {}^{O}\ddot{\mathbf{x}}_{OS} dt + \left(\delta\mathbf{R} - \mathbf{I}\right)\ {}^{O}\mathbf{g} - \frac{1}{\Delta t}\int_{t_1}^{t_2} \left(\delta\mathbf{R} - \mathbf{I}\right)\ {}^{O}\mathbf{g} dt \right\|_2 .\end{aligned} \tag{XVII}$$

Unlike δJ, δa does not depend on $\delta\dot{\mathbf{R}}$ and only on $\delta\mathbf{R}$. δa will be closed to one as long as the dynamic range of the reconstruction error signal $\Delta\mathbf{R}$ is close to 0, which implies that $\delta_{\delta\mathbf{R}}$ at different times points are similar to each other, i.e.

$$\delta\mathbf{R}\left(t_i\right)^{\top} \delta\mathbf{R}\left(t_j\right) \approx \mathbf{I}, \forall t_i, t_j \in [t_1, t_2]$$

When $\Delta_{\delta\mathbf{R}} \approx= 0 \implies \delta\mathbf{R} \approx \mathbf{R}$, where $\mathbf{R}$ is the fixed "average" orientation reconstruction error, then

$$\begin{aligned}{}^{O}\hat{\ddot{\mathbf{x}}}_{OS} &\approx \mathbf{R}\ {}^{O}\ddot{\mathbf{x}}_{OS} - \frac{1}{t_2 - t_1}\int_{t_1}^{t_2} \mathbf{R}\ {}^{O}\ddot{\mathbf{x}}_{OS} dt + \left(\mathbf{R} - \mathbf{I}\right)\ {}^{O}\mathbf{g} - \frac{1}{t_2 - t_1}\int_{t_1}^{t_2} \left(\mathbf{R} - \mathbf{I}\right)\ {}^{O}\mathbf{g} dt \\ &\approx \mathbf{R}\ {}^{O}\ddot{\mathbf{x}}_{OS} - \mathbf{0} + \left(\mathbf{R} - \mathbf{I}\right)\ {}^{O}\mathbf{g} - \left(\mathbf{R} - \mathbf{I}\right)\ {}^{O}\mathbf{g} = \mathbf{R}\ {}^{O}\ddot{\mathbf{x}}_{OS} \\ \implies \delta a &\approx 1\end{aligned} \tag{XVIII}$$

- **When ${}^{O}\ddot{\mathbf{x}}_{OS}$ is small** compared to gravity, δa can be large and is dominated by gravity.

$$\delta a \approx \frac{1}{\left\| {}^{O}\ddot{\mathbf{x}}_{OS} \right\|_2} \left\| \left(\delta\mathbf{R} - \mathbf{I}\right)\ {}^{O}\mathbf{g} - \frac{1}{\Delta t}\int_{t_1}^{t_2} \left(\delta\mathbf{R} - \mathbf{I}\right)\ {}^{O}\mathbf{g} dt \right\|_2 \tag{XIX}$$

- **Movements with larger acceleration reduce the effect of gravity**. Lets consider a movement with a larger amplitude than M, such that acceleration is $A\ {}^{O}\ddot{\mathbf{x}}_{OS}$, where $A > 1$ is the scale of the movement. Then, we have

$$\begin{aligned}\delta a_A &= \frac{1}{\left\| A\ {}^{O}\ddot{\mathbf{x}}_{OS} \right\|_2} \left\| \delta\mathbf{R}\ A\ {}^{O}\ddot{\mathbf{x}}_{OS} - \frac{A}{\Delta t}\int_{t_1}^{t_2} \delta\mathbf{R}\ {}^{O}\ddot{\mathbf{x}}_{OS} dt + \left(\delta\mathbf{R} - \mathbf{I}\right)\ {}^{O}\mathbf{g} - \frac{1}{\Delta t}\int_{t_1}^{t_2} \left(\delta\mathbf{R} - \mathbf{I}\right)\ {}^{O}\mathbf{g} dt \right\|_2 \\ &= \frac{1}{\left\| {}^{O}\ddot{\mathbf{x}}_{OS} \right\|_2} \left\| \delta\mathbf{R}\ {}^{O}\ddot{\mathbf{x}}_{OS} - \frac{1}{\Delta t}\int_{t_1}^{t_2} \delta\mathbf{R}\ {}^{O}\ddot{\mathbf{x}}_{OS} dt + \frac{\left(\delta\mathbf{R} - \mathbf{I}\right)\ {}^{O}\mathbf{g}}{A} - \frac{1}{A\ \Delta t}\int_{t_1}^{t_2} \left(\delta\mathbf{R} - \mathbf{I}\right)\ {}^{O}\mathbf{g} dt \right\|_2\end{aligned} \tag{XX}$$

  The contribution of the gravity terms to δa are reduced by a factor of A than that of Eq. XVII. A similar effect can be shown for faster movements.

Demonstration of the effects of reconstruction on LDLJ-A. We demonstrate the effect discussed in this section through a simple simulation. We simulated a minimum jerk movement of 30cm amplitude and 0.5s duration:

$${}^{O}\mathbf{x}_{OS} = 0.3\left(10\left(2t\right)^3 - 15\left(2t\right)^4 + 6\left(2t\right)^5\right)\mathbf{d}, \quad \text{where, } \mathbf{d} = \begin{bmatrix} d_x \\ d_y \\ d_z \end{bmatrix} \text{ and } \left\|\mathbf{d}\right\|_2 = 1,\ t \in \left[0, \frac{1}{2}\right]$$

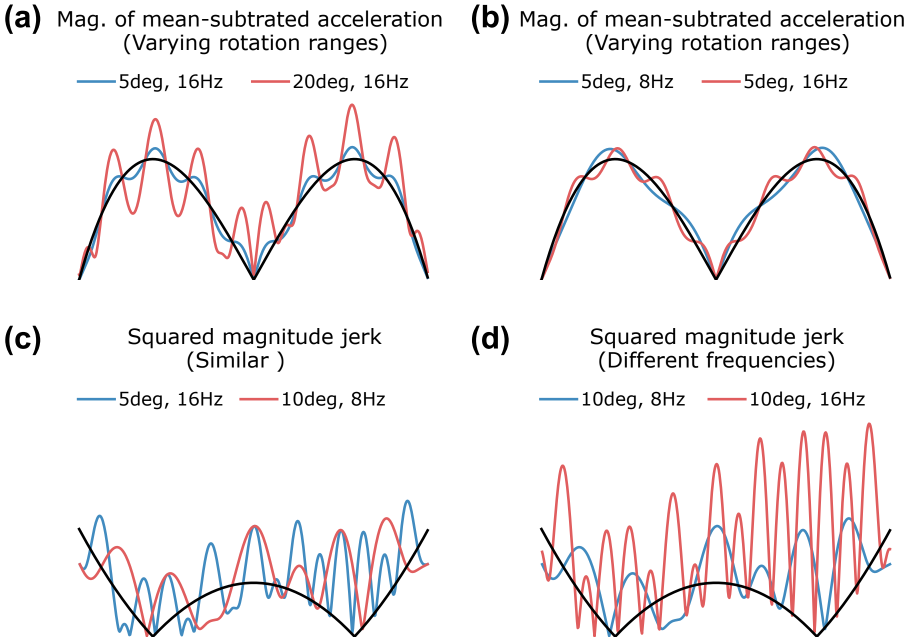

Figure IV: Effect of rotational error on the reconstructed kinematics: (a) effect of A_β on the magnitude of the mean subtracted reconstructed acceleration; (b) effect of f_β on the magnitude of the mean subtracted reconstructed acceleration; (c) effect of $\delta\dot{\mathbf{R}}$ on the jerk magnitude; and (d) larger $\delta\dot{\mathbf{R}}$ results in larger errors in the jerk magnitude. In all subplots, the black curve corresponds to the true movement.

The gravity vector was assumed to be ${}^{o}\mathbf{g} = \begin{bmatrix} 0 \\ 0 \\ -9.81 \end{bmatrix} m.s^{-2}$. The orientation reconstruction error was assumed to be a sinusoidal rotation about the y-axis:

$$\delta\mathbf{R}\left(t\right) = \mathbf{R}_y\left(\beta\left(t\right)\right) = \begin{bmatrix} \cos\beta\left(t\right) & 0 & \sin\beta\left(t\right) \\ 0 & 1 & 0 \\ -\sin\beta\left(t\right) & 0 & \cos\beta\left(t\right) \end{bmatrix} \qquad \beta\left(t\right) = A_\beta \sin\left(2\pi f_\beta t\right)$$

where

- A_β controls the dynamic range of the rotational error $\Delta_{\delta\mathbf{R}}$.
- A_β and f_β determined the rate of change of rotational error $\delta\dot{\mathbf{R}}\left(t\right)$.

The magnitude of the reconstructed acceleration is affected only by A_β and not f_β. On the other hand, the size of the errors in the jerk magnitude is related to the rate of change of orientation reconstruction error (Fig. IV(c)). Note that $\dot{\beta}\left(t\right)$ has the same peak value when $A_\beta = 5°, f_\beta = 16Hz$ and $A_\beta = 10°, f_\beta = 8Hz$. Finally, the error in the jerk magnitude increases with the rate of the change of orientation reconstruction error (Fig. IV(d)).

References

[1] S. Balasubramanian, A. Melendez-Calderon, A. Roby-Brami, and E. Burdet. On the analysis of movement smoothness. *Journal of NeuroEngineering and Rehabilitation*, 12(1):1–11, 2015.

[2] M. Yazdani, G. Gamble, G. Henderson, and R. Hecht-Nielsen. A simple control policy for achieving minimum jerk trajectories. *Neural Networks*, 27:74–80, 2012.
